# Incidence trends and specific risk factors of ischemic heart disease and stroke: An ecological analysis based on the Global Burden of Disease 2019

**DOI:** 10.1371/journal.pgph.0003920

**Published:** 2024-11-20

**Authors:** Ruiqi Xia, Min Cai, Ziyang Wang, Xuebo Liu, Jianfeng Pei, Maryam Zaid, Wanghong Xu

**Affiliations:** 1 Fudan University School of Public Health, Key Laboratory of Public Health Safety, Ministry of Education (Fudan University), Shanghai, China; 2 Yiwu Research Institute, Fudan University, Yiwu, Zhejiang Province, China; 3 Department of Cardiology, Shanghai Tongji Hospital, Tongji University School of Medicine, Shanghai, China; McMaster University, CANADA

## Abstract

Distribution of risk factors for cardiovascular diseases has been changing globally, which may account for the discrepant temporal trends of ischemic heart disease (IHD) and stroke. To test the hypothesis and identify potential contributing factors, we designed an ecological study based on the GDB-2019 data and extracted age-standardized incidence rates (ASIRs) of IHD and stroke, and summary exposure values (SEVs) of 87 attributable factors. A declining trend was observed for ASIRs of stroke (globally from 181.4 to 150.8/100,000 during 1990 and 2019, with an average annual percentage change of -0.64%) and IHD (globally from 316.4 to 262.4/100,000, with an average annual percentage change of -0.67%). However, the ASIR of IHD increased in Eastern Sub-Saharan Africa, Western Sub-Saharan Africa, East Asia, Central Asia, and Oceania, particularly in Uzbekistan and other 55 countries experiencing rapid socioeconomic translation. Eight factors, i.e. diet high in trans-fatty acids, diet low in calcium, high body-mass index, household air pollution from solid fuels, non-exclusive breastfeeding, occupational ergonomic factors, Vitamin A deficiency, and occupational exposure to particulate matter, gases, and fumes, were reversely associated with the ASIR of IHD and stroke at the country level. Ecological trend analysis also illustrated significant reverse associations of the factors with the ASIRs of IHD and stroke in in Western Sub-Saharan Africa, East Asia, and Oceania, but consistent associations in countries classified by the World Bank income levels. The results indicate the contributions of altered exposures to the eight factors in the discrepant trends of IHD and stroke across regions and countries, and suggest the determinant role of socioeconomic development in covariant of the risk factors with the incidences of IHD and stroke.

## Introduction

Cardiovascular disease (CVD) is a leading cause of death and the second contributor to disability worldwide, with ischemic heart disease (IHD) accounting for 16% and stroke for 11% of total deaths in 2019 [1]. Previous studies had observed declining incidences of stroke and IHD over past three decades [2]. However, the temporal trends varied greatly across regions. A systematic review demonstrated a decreasing or stabilizing worldwide trend in incidence of stroke, but an upward trend in some geographical areas of the Asian continent [3]. An ecological study based on the Global Burden of Disease (GBD) 2017 showed an increase in the incidence of stroke only among middle-income countries classified by the World Bank [4]. Meanwhile, a GBD study demonstrated variations in incidence trends of subtypes of CVD in specific regions or countries, including the increased IHD and the decreased stroke in China during 1990 and 2019 [5].

IHD and stroke are multi-factorial diseases related to environmental [6], behavioral [7], and cardiovascular metabolic factors [8]. Exposures to the risk factors have been changing, which may account for the discrepancies in temporal trends of incidences by subtypes of CVD. So far, however, little is known about the factors underlying the changing incidences. High systolic blood pressure, pollution exposure, smoking, and obesity have been associated with the risk of stroke [9, 10], while high systolic blood pressure, high low-density lipoprotein cholesterol and smoking were considered the major risk factors for IHD [11, 12]. The varied association magnitudes of the common risk factors with stroke and IHD and the altered distribution of the factors across populations may account for the contradicting trends in incidence of the two diseases in China and several other countries.

The GBD study provides us with comprehensive data to explore the specific risk factors for stroke and IHD at the population level. The GBD-2019 data has been used to estimate the global burden of overall and subtypes of CVD [11, 13]. However, no previous study has focused on the discrepant incidence trends of subtypes of CVD worldwide, nor explore the potential drivers underlying [5]. In this study, we compared the incidence and trends of IHD and stroke based on the GBD-2019, aiming to identify the potential risk factors accounting for the changing patterns in ecological perspective.

## Methods

### Data sources

The GBD-2019 provides accessible, up-to-date and comprehensive estimates of incidence, prevalence, mortality, years lived with disability (YLDs), years of life lost (YLLs), and disability adjusted Life Years (DALYs) for 369 diseases and injuries from 1990 to 2019 [14]. The diseases and injuries were presented as four-level mutually-exclusive “causes”, in which seven choices could be selected as the “causes” at level 1, twenty two at level 2 and more disaggregated “causes” at level 3 and 4 [14]. The data were available for 204 countries and territories under 7 super-regions and 21 regions.

The GBD-2019 also included 87 risk factors that were categorized broadly into three groups: 1) environmental and occupational, 2) behavioral, and 3) metabolic [15]. Summary exposure value (SEV) was used to summarize the exposure distribution of the risk factors at the global, regional and country levels. Detailed definitions of the risk factors and estimations of their SEVs have been described previously [16]. Briefly, the SEV is defined as a measure of a population’s exposure to a risk factor that takes into account the extent of exposure by risk level and the severity of that risk’s contribution to disease burden. SEV was calculated by the following formula:

$$SEV=\frac{\int_{x=l}^{u}RR(x)P(x)d(x)-1}{{RR}_{max}-1}$$

Where *RR(x)* is risk ratio at level *x* of exposure; *RR*_*max*_ is the highest risk ratio where more than 1% of population are exposed; *P(x)* is the density of exposure; and *l* and *u* are the lowest and the highest levels of exposure. The SEV is an effectively excess risk-weighted prevalence, which allows for comparisons across different types of exposures.

The methodology adopted by the GBD 2019 to estimate age-standardized incidence rates (ASIRs) of diseases and injuries, and SEVs of risk factors has been described previously [17]. Briefly, ASIRs were calculated to summarize the distribution of a disease or a factor at global, regional, and country levels. It was computed using the direct method involving multiplying the age-specific rates (*a*_*i*_), where *i* represented each age class, by the corresponding population count or weight (*w*_*i*_) for the same age subgroup in the GBD world standard population [18]. The products were summed and divided by the total of the standard population weights to normalize the rate across the population. The formula used was:

$$ASIR=\frac{\sum_{i=1}^{A}a_{i}w_{i}}{w_{i}}\times 100,000$$

The 95% uncertainty intervals (*UI*) were estimated as the 25^th^ and 975^th^ of 1,000 ordered draws from a bootstrap distribution. This calculation method adjusted the rates to account for different age distributions, enabling consistent comparisons across various populations.

### Data extraction

Then we extracted ASIRs of IHD, all type stroke, ischemic stroke (IS), intracerebral hemorrhage (ICH), and subarachnoid hemorrhage (SAH) at the national or territorial level from the Global Health Data Exchange (GHDx), source tool publically available (https://vizhub.healthdata.org/gbd-results/). Specifically, we used the GHDx tool and selected the terms of "non-infectious diseases" as the level 1 “causes”, “cardiovascular diseases” as the level 2 “causes”, "IHD " and "stroke" as the level 3 “causes”, and finally “IS”, “ICH”, and “SAH” as the level 4 “causes”, and the term of "Incidence" as the "measure". IHD was coded as I20-I25 according to the 10^th^ revision of the International Classification of Diseases (ICD-10), while all type stroke, IS, ICH and SAH were coded as I60-69, I63, I61 and I60, respectively.

The SEVs of 87 risk factors at four levels were also derived from the website for all 204 countries and territories. A total of 67 most detailed factors were included in this analysis, including 27 environmental and occupational factors, 34 behavior and 6 metabolic factors.

To obtain sex- and age-specific data, we extracted data by selecting the terms of "Male", "Female" and "Both" for gender, and the term of "age-standardized" for age. Considering the wide disparity in incidence of CVD between people under or above 70 years [19], and the fact that the World Health Organization defined "premature death" as death at ages less than 70 years [20], we also extracted the crude incidence by selecting "<70 years" and "70+ years" for age.

### Statistical analysis

Join-point regression was applied to profile the temporal trends of ASIRs of IHD and stroke and SEVs of risk factors by sex, regions, and countries [21]. With a linear regression model, the average annual percentage change (AAPC) and 95% confidence interval (CI) were calculated as the weighted average of annual percentage change to evaluate the change in rates from 1990 to 2019.

Pearson correlation analysis was applied at the country level to estimate the correlation coefficients (r) of SEVs of risk factors with ASIRs of IHD and stroke, based on which we identified the factors significantly related with IHD and stroke. As shown in **S1 Fig in S1 File**, eight of 87 factors were consistently contradictorily correlated with the ASIRs of IHD and stroke in the analyses using the data of 1990, 2000, 2010 and 2019, and thus selected as potential contributors for the different changing patterns of the two diseases in incidence, which included diet high in trans-fatty acids, diet low in calcium, high body-mass index, household air pollution from solid fuels, non-exclusive breastfeeding, occupational ergonomic factors, occupational particulate matter, gases, and fumes, and Vitamin A deficiency. All these factors have been associated with the risk of CVD [22–31].

Based on the country-level data in 2019, the linear shape of the relationship between SEVs of selected factors and ASIRs of IHD and stroke were determined using locally weighted scatter plot smoothing (LOWESS) technique. Then linear regression model was applied to estimate respective beta coefficient (β) and 95%CI. Stratified analyses were performed to evaluate the associations across subgroups. Ecological trend analysis was further applied to evaluate the associations of selected risk factors with ASIRs of IHD and stroke over 1990 and 2019 using Bayesian model to deal with the time-series data [32]. The models were fitted by setting the number of chains to three (n.chains = 3), with 10,000 iterations for each chain (n.iter = 10,000), and discarding the first 1,000 iterations as burn-in (n.burnin = 1,000). The random seed number of 123 was selected to ensure the data reproducibility.

Data analyses were performed using R 4.2.0 packages, which included “magrittr”, “plyr”, “dplyr”, “data.table”, “ggplot2”, “epitools”, “tidyverse”, “maps”, “RColorBrewer”, “readxl”, “pheatmap”, “ggalt”, “lemon”, “patchwork”, “ggtext”, “cowplot”, “ggeasy”, “rjags” and “R2jags”. Joinpoint Regression Program 4.9.1.0 was used to evaluate secular trends. *P* value less than 0.05 was considered statistically significant.

## Results

### Trends of IHD and strokes

#### Age-standardized incidence and temporal trends of IHD

**Fig 1:**
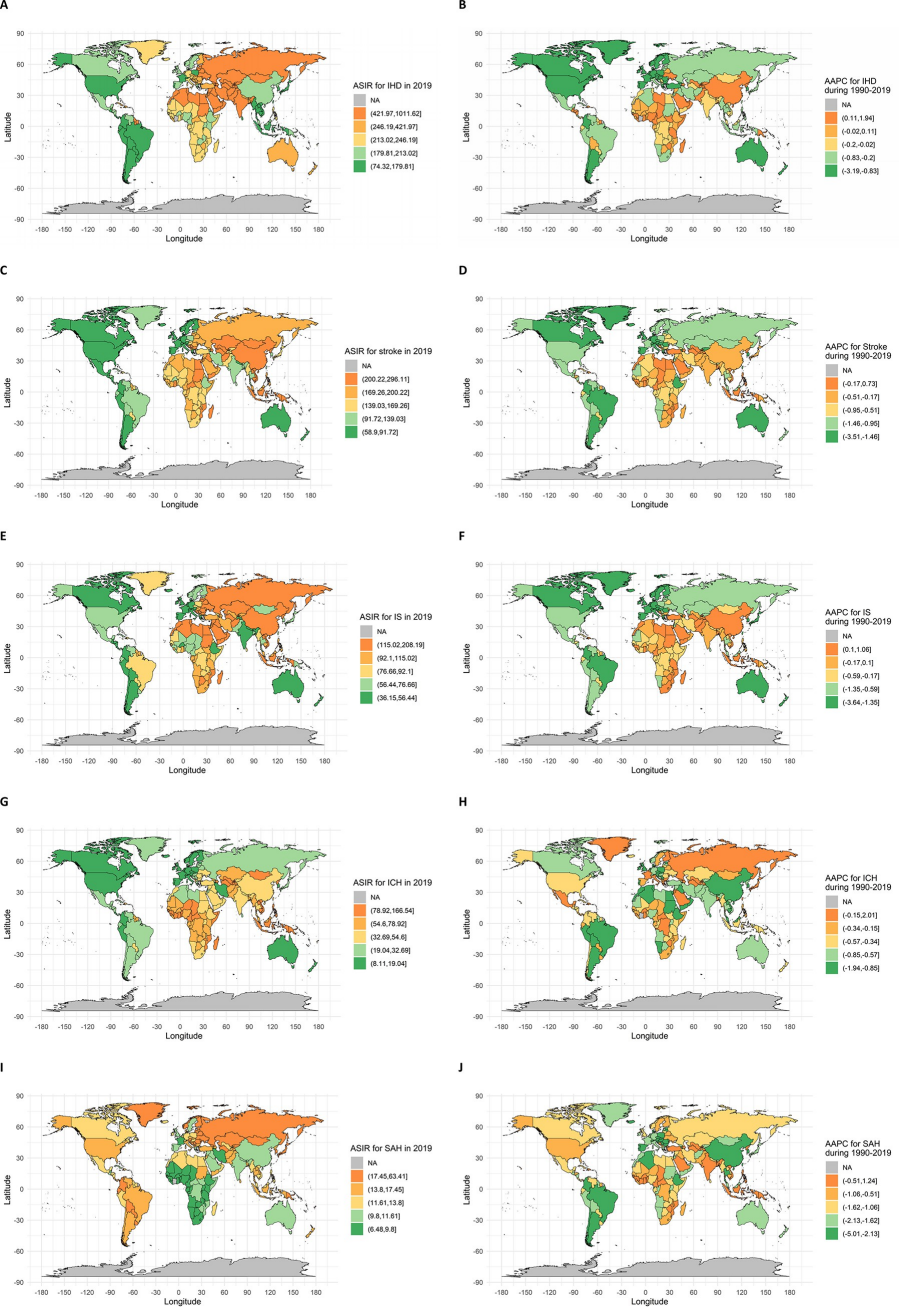
Age-standardized incidence (1/100,000) of cardiovascular diseases in 2019 (left) and AAPC during 1990 and 2019 (right) by countries or territories. Ischemic heart disease (A), ischemic stroke (B), intracerebral hemorrhage(C) and subarachnoid hemorrhage (D). Note: the base layer of the maps were profiled by using R package of “maps”, which referred to the website of https://gadm.org/index.html.

Globally, the ASIR of IHD decreased from 316.4 (282.2, 352.3) to 262.4 (233.3, 293.3) per 100,000 during 1990 and 2019, with an AAPC of -0.67% (-0.79, -0.56) over the decades. The declining trend was observed in both sexes, leading to a higher ASIR in men (333.5/100,000, 95%UI: 297.0, 371.9) than in women (198.5/100,000, 95%UI: 176.4, 221.2) in 2019. The incidence of IHD varied from 78.6/100,000 in Andean Latin America to 652.4/100,000 in Central Asia in 2019, and declined in most regions, particularly the high-income North America (AAPC: -2.64%, 95%CI: -2.72, -2.57), Western Europe (AAPC: -1.59%, 95%CI: -1.62, -1.56), Central Europe (AAPC: -1.51%, 95%CI: -1.89, -1.13), and Southern Latin America (AAPC: -1.31%, 95%CI: -1.43, -1.18). However, the ASIR of IHD was found to increase in East Asia, Central Asia, Western Sub-Saharan Africa, and Eastern Sub-Saharan Africa, with an AAPC of 0.34% (0.27, 0.42), 0.26% (0.15, 0.36), 0.17% (0.14, 0.20), and 0.07% (0.06, 0.09), respectively **(Table 1)**. At the country level, the ASIR of IHD was 1011.6/100,000 (952.1, 1078.8) in Uzbekistan in 2019, ranking the first globally and being almost 14-fold of the incidence in Peru (**Fig 1**). The increase in ASIR of IHD from 1990 to 2019 was also the highest in Uzbekistan, with an AAPC of 1.94%, followed by Tajikistan (0.83%), Azerbaijan (0.59%) and Pakistan (0.48%). Parallel to the upward trend of IHD in 77 countries were the decreasing trend in 126 countries/territories, with the largest AAPC of -3.19% (-3.66, -2.73) in Poland.

**Table 1:** Age-standardized incidences (1/100,000) of ischemic heart disease and all type stroke and AAPCs during 1990 to 2019.

	ASIR (95%UI) of IHD		AAPC (95%CI)	ASIR (95%UI) of all type stroke		AAPC (95%CI)
	1990	2019		1990	2019	
**Global**	316·4 (282·2,352·3)	262·4 (233·3,293·3)	-0·67 (-0·79,-0·56)	181·4 (165·2,199·8)	150·8 (136·5,167·5)	-0·64 (-0·67,-0·62)
**By Sex**						
Male	405·3 (361·2,452·1)	333·5 (297·0,371·9)	-0·70 (-0·82,-0·57)	178·5 (162·7,196·8)	151·1 (136·9,167·5)	-0·58 (-0·62,-0·55)
Female	239·9 (213·1,267·5)	198·5 (176·4,221·2)	-0·68 (-0·78,-0·58)	182·0 (165·7,201·3)	149·8 (135·6,166·6)	-0·68 (-0·71,-0·65)
**By region**						
Eastern Sub-Saharan Africa	199·13(173·3,227·2)	203·2 (177·8,231·4)	0·07 (0·06,0·09)	198·7 (183·6, 216·8)	157·8 (145·1, 172·6)	-0·80 (-0·83,-0·77)
Western Sub-Saharan Africa	208·5 (180·4,238·4)	219·4 (191·0,250·2)	0·17 (0·14,0·20)	177·9 (164·1, 194·2)	158·9 (146·9, 173·4)	-0·39 (-0·41,-0·37)
Southern Sub-Saharan Africa	255·7 (222·1,292·1)	244·1 (213·0,278·5)	-0·15 (-0·18,-0·13)	166·9 (150·3, 186·8)	163·6 (145·1, 186·3)	-0·07 (-0·11,-0·03)
Central Sub-Saharan Africa	236·8 (206·2,267·5)	227·7 (200·5,256·1)	-0·14 (-0·15,-0·12)	194·5 (180·2, 211·3)	162·3 (149·8, 176·6)	-0·63 (-0·65,-0·61)
North Africa and Middle East	674·5 (612·2,740·5)	613·9 (555·8,675·2)	-0·33 (-0·37,-0·30)	193·4 (177·8, 211·0)	183·0 (166·7, 201·7)	-0·20 (-0·21,-0·18)
Caribbean	391·5 (345·7,438·7)	366·5 (323·0,412·6)	-0·20 (-0·30,-0·11)	142·0 (132·5, 153·2)	129·5 (119·8, 140·1)	-0·32 (-0·33,-0·30)
Central Latin America	206·9 (181·7,233·8)	187·3 (165·1,211·6)	-0·36 (-0·43,-0·29)	133·4 (122·0, 146·5)	91·6 (83·6, 100·7)	-1·29 (-1·33,-1·25)
Southern Latin America	236·3 (214·6,259·7)	162·7 (145·2,182·5)	-1·31 (-1·43,-1·18)	138·9 (129·2, 149·5)	90·0 (83·1, 97·4)	-1·49 (-1·53,-1·44)
Andean Latin America	85·2 (74·5,96·5)	78·6 (68·7,88·6)	-0·35 (-0·61,-0·08)	115·2 (107·1, 124·2)	87·9 (80·8, 95·6)	-0·93 (-0·95,-0·91)
Tropical Latin America	124·6 (109·6,139·9)	109·7 (96·8,123·3)	-0·51 (-0·76,-0·26)	223·3 (200·5, 250·1)	127·3 (114·2, 142·3)	-1·93 (-2·01,-1·86)
High-income North America	381·6 (333·0,435·5)	177·7 (162·2,195·2)	-2·64 (-2·72,-2·57)	117·6 (103·4, 134·6)	84·8 (75·5, 95·5)	-1·11 (-1·20,-1·03)
High-income Asia Pacific	155·3 (135·8,175·7)	122·0 (106·4,138·4)	-0·83 (-0·98,-0·68)	187·7 (168·6, 210·7)	133·0 (120·4, 147·2)	-1·18 (-1·20,-1·17)
East Asia	177·0 (156·7,199·0)	195·9 (174·6,220·7)	0·34 (0·27,0·42)	222·3 (198·1, 249·9)	200·6 (177·0, 230·2)	-0·37 (-0·50,-0·24)
South Asia	425·8 (371·4,481·9)	427·6 (374·9,480·9)	-0·02 (-0·19,0·14)	130·6 (118·3, 145·0)	117·3 (105·9, 130·4)	-0·37 (-0·41,-0·34)
Southeast Asia	147·5 (130·3,166·3)	135·9 (121·0,151·4)	-0·34 (-0·56,-0·12)	232·6 (213·7, 255·4)	215·9 (197·8, 238·2)	-0·25 (-0·26,-0·24)
Central Asia	599·0 (542·3,660·0)	652·4 (600·2,709·4)	0·26 (0·15,0·36)	233·1 (217·9, 250·7)	195·2 (182·9, 208·8)	-0·60 (-0·65,-0·56)
Eastern Europe	535·2 (473·4,600·7)	514·4 (455·6,579·4)	-0·18 (-0·33,-0·02)	261·1 (231·6, 295·1)	192·0 (171·5, 216·2)	-1·05 (-1·08,-1·02)
Central Europe	384·3 (348·4,422·5)	253·7 (228·8,277·9)	-1·51 (-1·89,-1·13)	230·6 (211·7, 253·0)	150·7 (137·2, 165·8)	-1·46 (-1·48,-1·43)
Western Europe	325·3 (296·5,356·3)	205·0 (182·9,227·2)	-1·59 (-1·62,-1·56)	120·8 (110·2, 133·6)	69·8 (63·5, 76·6)	-1·88 (-1·93,-1·82)
Oceania	203·3 (176·5,231·9)	209·1 (183·4,237·3)	0·08 (-0·01,0·17)	232·2 (217·8, 248·8)	216·5 (202·4, 232·2)	-0·24 (-0·25,-0·24)
Australasia	479·2 (431·2,530·4)	345·2 (304·1,388·8)	-1·11 (-1·19,-1·04)	109·1 (99·7, 119·8)	65·1 (59·0, 71·8)	-1·77 (-1·81,-1·73)

*ASIR: Age-standardized incidence rate; AAPC: Average annual percentage change; UI: Uncertainty Interval; CI: Confidence Interval

### Age-standardized incidence and temporal trends of stroke

Globally, the ASIR of stroke decreased from 181.4 (165.2, 199.8) to 150.8 (136.5, 167.5) per 100,000 during 1990–2019, with an AAPC of -0.64% (-0.67, -0.62). The incidence was higher in men (151.1 per 100,000, 95%UI: 136.9, 167.5) than in women (149.8 per 100,000, 95%UI: 135.6, 166.6) in 2019. The highest ASIR was observed in Oceania (216.5 per 100,000, 95%UI: 202.4, 232.2). All regions experienced a decrease in ASIR of stroke over the decades, with the most significant decline in Tropical Latin America (AAPC: -1.93%, 95%CI: -2.01, -1.86) (**Table 1**). At the country level, Kiribati had the highest incidence of stroke (296.1 per 100,000, 95%UI: 278.5, 316.6), while Zimbabwe experienced the highest increase in incidence of stroke over the past three decades (AAPC: 0.73%, 95%CI: 0.65, 0.81) (**Fig 1**). Overall, 109 of 204 countries demonstrated decreasing ASIRs of both IHD and stroke, and 56 with decreasing stroke but increasing IHD.

A global decreasing trend in ASIR was observed for all subtypes of stroke during 1990 and 2019, with a slower decline in ASIR of IS (AAPC: -0.36%, 95%CI: -0.38, -0.34) than in ASIRs of ICH (AAPC: -1.20%, 95%CI: -1.31, -1.09) and SAH (AAPC: -0.63%, 95%CI: -0.71, -0.56) **(S2 Table in S1 File)**. However, the ASIR of IS was found to increase in Southern Sub-Saharan Africa, North Africa and Middle East, East Asia and Southeast Asia, while the ASIR of SAH increased in high-income Asia Pacific. Presented in **Fig 1** are the incidence trends of the subtypes at the countries/territories level. The ASIR of IS increased in 48 countries, but decreased in 156 countries, with Egypt experiencing the most increase (AAPC: 1.06%, 95%CI: 1.02, 1.11), and Portugal showing the most decrease (AAPC: -3.64%, 95%CI: -3.77, -3.51). For ICH, an increasing trend was observed in 8 countries, and a decreasing trend in 196 countries, with Saint Vincent and the Grenadines increasing most (AAPC: 1.24%, 95%CI: 1.17, 1.32), and the Republic of Korea decreasing most (AAPC: -5.01%, 95%CI: -5.18, -4.84). For SAH, an upward incidence trend was found in 24 countries, while a decreasing trend in 180 countries, with the most increase in Japan (AAPC: 2.01%, 95%CI: 1.85, 2.17) and the most decrease in Republic of Korea (AAPC: -1.94%, 95%CI: -2.12, -1.76).

### Trends of IHD and stroke by age, sex and world bank income level

As shown in S1 Fig in S1 File, the crude incidence of IHD and three subtypes of stroke increased among people less than 70 years, the candidate group for premature death defined by the World Health Organization for both sexes, but decreased among those aged 70 years or above during 1990 and 2019. Further stratified analysis demonstrated decreasing crude (S2 Fig in S1 File) and age-specific incidences of IHD and stroke across subgroups (S3 Fig in S1 File) in both men and women.

The trends in ASIR of IHD and stroke by the World Bank income levels are displayed in **S4 Fig in S1 File**. Over the period of 1990 and 2019, the incidence of IHD was the highest in the lower-middle income countries, while the incidence of IS ranked the first in the upper-middle income countries, and the incidence of ICH in the low-income countries. Regardless of sex, the incidence of IHD remained stable in lower-middle, upper-middle, and low-income countries, but decreased rapidly in high-income countries. A similar rapid decrease was observed for ASIR of IS and ICH in high-income countries, but an increasing trend was found for ASIR of SAH.

### Selected risk factors and their associations with IHD and stroke

As presented in **S3 Table in S1 File**, while the SEV of high body mass index increased globally during 1990 and 2019 (AAPC: 1.96%, 95%CI: 1.93, 1.98), the SEVs of the other seven factors decreased significantly over the period. The temporal trends differed by sex in the SEV of occupational particulate matter, gases, and fumes, but were consistent for other factors. When classified by the World Bank income levels, the changing trends in each risk factor were consistent within the countries with high, upper-middle, lower-middle or low income levels, with a higher AAPC for almost all factors in the countries with a low income level **(S4 Table in S1 File**).

**Fig 2:**
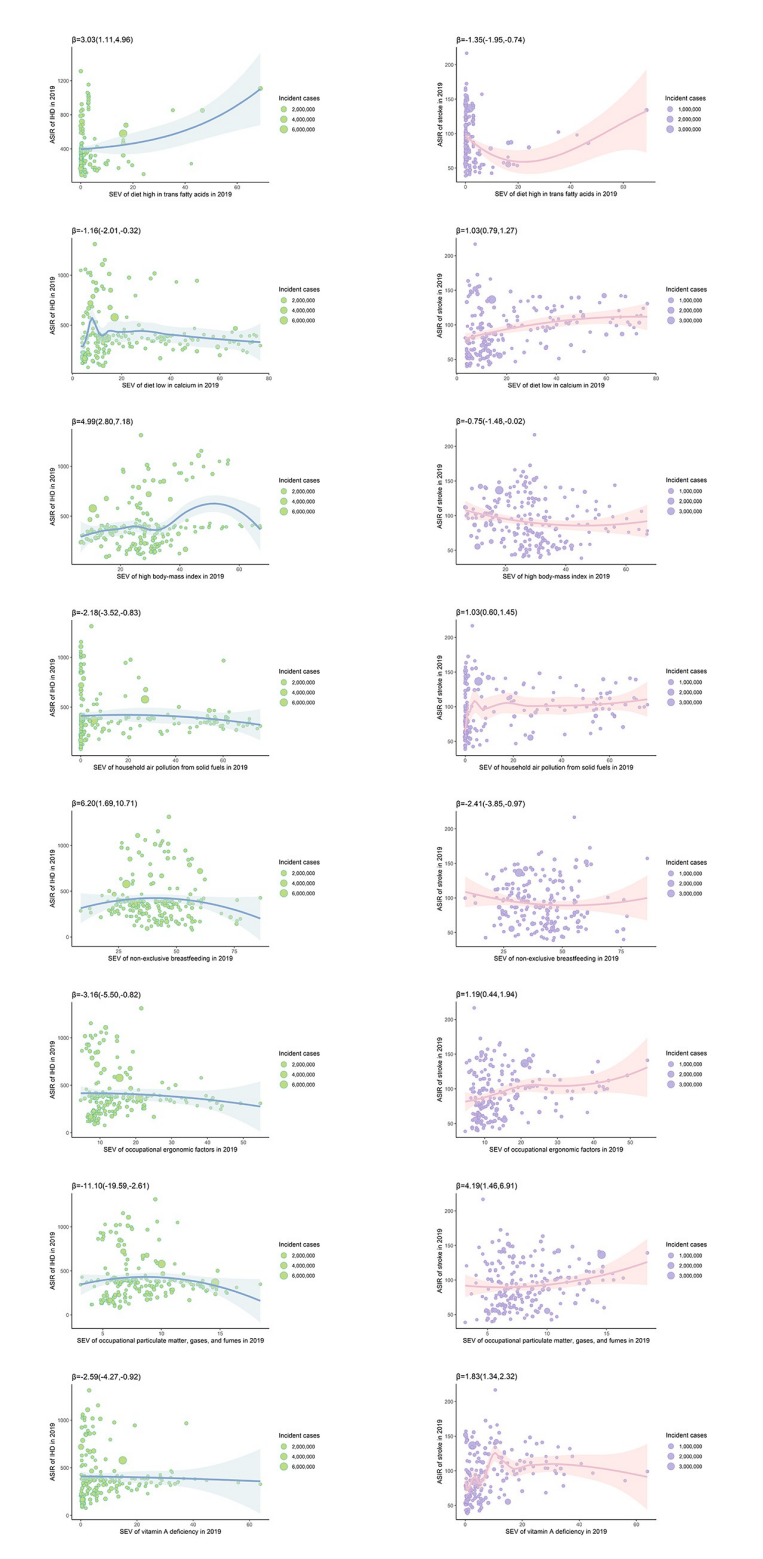
Associations of SEVs (%) of selected risk factors with ASIR (1/100,000) of ischemic heart disease and all type stroke in 2019 at the country level.

**Fig 3:**
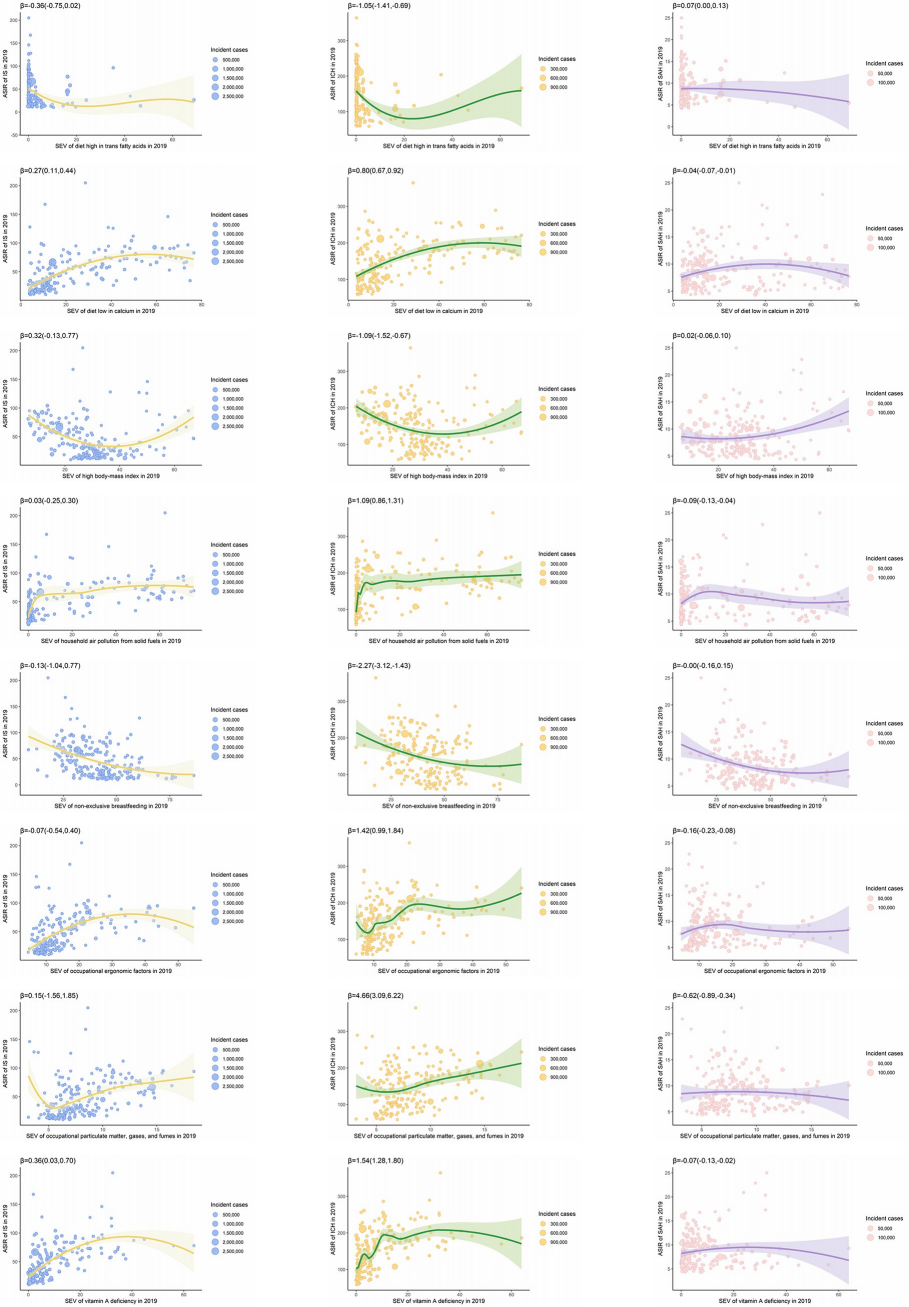
Associations of SEVs (%) of selected risk factors with ASIR (1/100,000) of subtypes of stroke in 2019 at the country level.

Diet high in trans-fatty acids, high body mass index, and non-exclusive breastfeeding were positively associated with ASIR of IHD in 2019 at the country level, with β (95%CI) of 3.03 (1.11, 4.96), 4.99 (2.80, 7.18) and 6.20 (1.69, 10.71), respectively, while the other five factors were inversely associated with the disease (**Fig 2)**. The associations of the factors with all type stroke were contradictory to those with IHD, with β (95%CI) being -1.35 (-1.95,-0.74) for diet high in trans-fatty acids, -0.75 (-1.48, -0.02) for high body mass index, -2.41 (-3.85,-0.97) for non-exclusive breastfeeding, 1.03 (0.79,1.27) for diet low in calcium, 1.03 (0.60, 1.45) for household air pollution from solid fuels, 1.19 (0.44,1.94) for occupational ergonomic factors, 4.19 (1.46, 6.91) for occupational particulate matter, gases and fumes, and 1.83(1.34. 2.32) for Vitamin A deficiency (all p values for β < 0.05). Most of the factors were not associated with the ASIR of IS at the country level, whereas the associations of the factors with ICH were contradictory to those with SAH, as shown in **Fig 3**.

### Ecological trend analyses for selected risk factors with ASIR of IHD and stroke

Ecological trend analyses at the global level revealed significant negative associations of high body mass index with ASIR of IHD and stroke, and significant positive associations of other factors with IHD and all type stroke over the period of 1990 and 2019 (**Table 2**). A similar association pattern was observed for stroke in almost all regions, and for IHD in most regions but Western Sub-Saharan Africa, East Asian, and Oceania, in which a positive association was found for high body mass index, and negative associations for other factors.

**Table 2:** Ecological trend analysis for SEV (%) of selected risk factors with ASIR (1/100,000) of IHD and stroke during 1990 and 2019 at the global and region levels.

	β coefficient (95% Confidence Interval)							
	High body mass index	Vitamin A deficiency	Non-exclusive breastfeeding	Diet high in trans-fatty acids	Diet low in calcium	Occupational ergonomic factors	Occupational particulate matter, gases & fumes	Household air pollution from solid fuels
**IHD**								
Global	-8·2 (-8·8, -7·7)	3·5 (3·1, 3·8)	23·0 (19·4, 25·2)	7·3 (6·5, 8·1)	8·9 (7·7, 9·7)	14·2 (12·9, 15·3)	72·5 (20·3, 99·5)	4·3 (4·0, 4·5)
By region								
Eastern Sub-Saharan Africa	-0·3 (-0·5, 0·0)	0·1 (0·0, 0·1)	0·3 (-0·1, 0·6)	0·6 (0·0, 4·2)	0·2 (-0·1, 1·3)	0·1 (-0·1, 0·3)	1·3 (-6·9, 13·8)	0·1 (0·0, 0·1)
Western Sub-Saharan Africa	0·6 (0·4, 0·9)	-0·2 (-0·3, -0·1)	-1·6 (-2·3, -1·0)	-1·2 (-2·3, 1·2)	-0·4 (-0·6, -0·2)	-0·1 (-0·6, 0·4)	10·5 (-10·3, 41·6)	-0·2 (-0·4, -0·1)
Southern Sub-Saharan Africa	-1·8 (-2·2, -1·5)	1·0 (0·8, 1·3)	3·1 (2·8, 3·4)	16·0 (-6·5, 27·9)	3·9 (-3·6, 7·1)	-8·8 (-14·5, 14·3)	14·0 (10·8, 17·4)	1·4 (1·0, 1·7)
Central Sub-Saharan Africa	-3·8 (-4·9, -2·8)	0·6 (0·4, 0·8)	1·9 (1·8, 2·1)	4·6 (3·8, 11·7)	3·1 (-0·5, 9·4)	2·0 (1·2, 2·8)	29·6 (-8·7, 81·9)	0·6 (0·6, 0·7)
North Africa and Middle East	-6·6 (-7·1, -6·0)	6·2 (5·5, 6·8)	21·9 (19·7, 23·9)	13·5 (6·5, 15·8)	6·8 (6·6, 7·1)	28·9 (14·9, 40·6)	95·9 (76·1, 99·7)	10·8 (10·1, 11·6)
Caribbean	-3·9 (-4·5, -3·2)	3·7 (3·1, 4·3)	9·0 (7·6, 10·6)	5·8 (4·9, 7·3)	4·0 (3·1, 5·8)	-18·1 (-25·5, -6·4)	66·9 (2·9, 85·3)	6·0 (5·3, 6·7)
Central Latin America	-2·8 (-3·0, -2·6)	1·9 (1·8, 2·1)	13·6 (7·5, 20·6)	1·3 (1·2, 1·5)	2·2 (2·0, 2·4)	9·8 (9·1, 10·7)	42·8 (1·9, 54·3)	2·9 (2·6, 3·1)
Southern Latin America	-7·7 (-8·3, -7·2)	9·7 (8·3, 11·0)	34·4 (2·1, 79·6)	14·7 (9·5, 17·8)	13·8 (8·5, 19·7)	-34·0 (-52·8, -19·6)	98·1 (74·2, 99·9)	29 (27·2, 30·9)
Andean Latin America	-0·9 (-1·2, -0·5)	0·7 (0·4, 0·9)	1·7 (1·4, 2·0)	0·9 (0·3, 2·0)	0·6 (0·4, 0·8)	-1·6 (-2·1, -1·2)	28·4 (-11·4, 87·6)	0·9 (0·6, 1·2)
Tropical Latin America	-0·8 (-1·1, -0·5)	0·7 (0·5, 1·0)	2·8 (2·3, 3·4)	0·9 (0·4, 1·3)	0·7 (0·5, 0·8)	-3·3 (-4·7, -1·7)	25·2 (12·8, 32·4)	1·4 (1·1, 1·7)
High-income North America	-16·5 (-18·3, -14·9)	93·6 (68·6, 99·8)	32·8 (19·4, 44·4)	22·5 (10·2, 34·7)	-1·8 (-40·0, 33·3)	96·0 (78·0, 99·8)	93·7 (66·6, 99·8)	14·5 (-93·1, 95·9)
High-income Asia Pacific	-12·4 (-13·9, -11·0)	30·8 (24·8, 36·8)	31·5 (5·3, 39·8)	14·1 (4·2, 17·1)	0·6 (-4·7, 17·6)	24·9 (21·9, 27·8)	41·7 (34·9, 47·3)	94·3 (68·1, 99·8)
East Asia	3·5 (2·7, 4·4)	-2·1 (-2·5, -1·7)	-6·0 (-7·2, -4·7)	-3·4 (-3·9, -3·0)	-1·4 (-1·7, -1·1)	-2·0 (-2·3, -1·6)	-13·4 (-25·9, 17·4)	-1·1 (-1·3, -0·9)
South Asia	-1·7 (-3·8, 0·6)	0·3 (-0·1, 0·7)	2·4 (-2·8, 7·7)	0·7 (-0·4, 1·6)	2·0 (0·5, 3·6)	3·7 (1·0, 6·0)	25·5 (-12·9, 41·8)	0·3 (-0·1, 0·8)
Southeast Asia	-2·3 (-2·9, -1·7)	0·8 (0·5, 1·0)	5·8 (4·5, 7·2)	7·4 (5·4, 12·6)	1·8 (1·2, 2·5)	-2·4 (-9·7, 7·8)	31·3 (-1·6, 55·5)	0·9 (0·6, 1·2)
Central Asia	-3·4 (-6·8, 0·1)	2·0 (-2·5, 6·5)	1·8 (-5·1, 13·1)	3·0 (-1·2, 6·6)	3·2 (1·1, 5·0)	4·7 (-6·9, 13·5)	3·5 (-71·3, 61·4)	-0·2 (-4, 3·9)
Eastern Europe	-10·0 (-13·5, -7·1)	86·8 (51·7, 99·4)	44·0 (13·7, 68·6)	10·4 (7·1, 14·6)	7·4 (5·4, 9·6)	-7·7 (-36·8, 18·6)	75·6 (41·5, 98·2)	43·1 (16·8, 70·1)
Central Europe	-19·4 (-20·8, -18·1)	15·5 (14·3, 16·7)	54·9 (7·2, 98·8)	12·0 (11·0, 13·0)	28·1 (24·4, 31·9)	44·7 (40·1, 48·6)	98·8 (94·3, 100·0)	31·9 (27·9, 35·8)
Western Europe	-17·6 (-18·1, -17·0)	83·1 (67·3, 97·4)	63·5 (21·4, 78·3)	11·3 (9·7, 12·7)	50·2 (45·1, 56·0)	64·1 (2·0, 98·3)	97·9 (89·0, 99·9)	81·2 (-1·0, 99·2)
Oceania	1·2 (0·7, 1·9)	-0·1 (-0·4, 0·2)	-2·5 (-3·8, 6·5)	-0·9 (-1·4, 1·4)	4·6 (-2·8, 6·0)	1·0 (0·7, 1·4)	-4·9 (-9·3, 9·6)	-0·3 (-0·6, -0·1)
Australasia	-12·0 (-12·4, -11·6)	27·0 (-90·8, 96·8)	38·4 (-36·0, 88·7)	8·6 (6·8, 10·0)	-4·7 (-22·9, 9·6)	-78·9 (-99·4, -16·9)	97·3 (87·4, 99·9)	80·1 (-8·7, 99·1)
**All type stroke**								
Global	-4·9 (-5·3, -4·5)	2·1 (1·8, 2·3)	13·7 (10·3, 16·0)	4·4 (4·0, 4·8)	5·4 (4·4, 6·1)	8·4 (7·6, 9·2)	35·7 (3·7, 54·5)	2·6 (2·4, 2·7)
By region								
Eastern Sub-Saharan Africa	-6·8 (-8·0, -5·6)	1·8 (1·5, 2·0)	10·5 (9·7, 11·3)	14·2 (12·0, 16·4)	4·8 (3·4, 5·3)	5·1 (4·8, 5·5)	40·5 (21·9, 48·3)	2·3 (2·0, 2·5)
Western Sub-Saharan Africa	-2·6 (-2·8, -2·4)	0·9 (0·8, 0·9)	5·9 (4·9, 6·8)	7·2 (5·0, 16·7)	1·6 (1·6, 2·9)	2·8 (1·9, 4·0)	25·8 (-2·0, 52·6)	1·0 (1·0, 1·1)
Southern Sub-Saharan Africa	-1·1 (-2·3, 0·0)	0·6 (-0·1, 1·3)	3·7 (2·2, 4·8)	8·8 (-2·8, 14·9)	2·5 (-2·8, 4·8)	2·9 (-4·0, 18·2)	8·0 (1·2, 15·2)	0·7 (-0·2, 1·7)
Central Sub-Saharan Africa	-8·1 (-11·4, -4·9)	1·1 (0·6, 1·7)	4·6 (4, 5·3)	11·1 (8·8, 13·2)	0·4 (-3·0, 7·7)	3·6 (1·2, 5·7)	1·7 (-30·3, 30·4)	1·5 (1·1, 1·8)
North Africa and Middle East	-0·9 (-1·1, -0·8)	0·9 (0·7, 1·0)	3·3 (2·7, 4·2)	1·8 (-0·1, 4·3)	1·0 (0·8, 1·1)	3·4 (0·9, 6·2)	14·4 (9·8, 27·5)	1·6 (1·4, 1·8)
Caribbean	-1·8 (-2·2, -1·4)	1·8 (1·5, 2·1)	4·8 (4·3, 7·3)	2·9 (2·2, 3·8)	1·9 (1·2, 3·1)	-11·1 (-13·6, -0·3)	44·3 (19·8, 84·7)	3·0 (2·7, 3·3)
Central Latin America	-5·0 (-5·4, -4·5)	3·4 (3·1, 3·7)	22·2 (12·9, 24·7)	2·3 (2·0, 2·7)	4·0 (3·9, 4·2)	17·0 (15·0, 18·9)	74·7 (18·2, 96·5)	5·1 (4·6, 5·5)
Southern Latin America	-5·0 (-5·3, -4·7)	6·3 (5·5, 7·2)	31·5 (17·1, 57·7)	10·7 (8·9, 12·1)	8·4 (5·2, 11·0)	-25·3 (-35·3, -15·6)	89·9 (41·7, 99·3)	18·6 (17·2, 20·1)
Andean Latin America	-2·5 (-2·8, -2·2)	2·0 (1·8, 2·2)	4·0 (3·5, 4·5)	3·0 (2·5, 3·6)	1·7 (1·6, 2·0)	-4·4 (-4·7, -4·0)	-1·8 (-18·9, 45·7)	2·5 (2·3, 2·7)
Tropical Latin America	-6·0 (-6·4, -5·5)	5·6 (5·2, 6·0)	13·8 (10·4, 17·1)	8·0 (6·5, 9·5)	4·5 (4·3, 4·7)	-23·1 (-31·5, -14·4)	98·5 (70·4, 100)	8·8 (8·4, 9·2)
High-income North America	-2·4 (-2·6, -2·3)	32·8 (24·9, 40·7)	3·9 (1·9, 6·2)	3·9 (2·6, 5·0)	1·9 (-1·8, 6·1)	28·8 (24·1, 43·4)	46·2 (35·8, 68·7)	68·4 (-49·6, 98·9)
High-income Asia Pacific	-15·2 (-18·0, -12·7)	39·4 (31·2, 47·6)	38·0 (5·7, 43)	16·0 (2·3, 23·7)	2·6 (-4·2, 9·6)	28·6 (22·2, 34·4)	57·5 (53·3, 60·2)	91·9 (57·3, 99·7)
East Asia	-5·1 (-5·9, -4·3)	2·7 (2·1, 3·3)	8·1 (6·8, 9·8)	4·8 (4·5, 5·1)	2·0 (1·6, 2·3)	2·7 (2·4, 3·0)	34·4 (7·7, 44·2)	1·4 (1·1, 1·7)
South Asia	-2·8 (-3·3, -2·2)	0·5 (0·5, 0·6)	-1·6 (-5·3, 2·7)	1·2 (1·0, 1·5)	1·7 (0·9, 2·3)	3·4 (2·4, 4·5)	40·7 (10·4, 74·8)	0·6 (0·5, 0·7)
Southeast Asia	-2·4 (-2·6, -2·3)	0·9 (0·8, 1·0)	5·8 (4·9, 7·0)	7·8 (3·9, 17·1)	1·9 (1·4, 3·2)	0·2 (-7·3, 17·6)	37·0 (1·6, 73·3)	1·1 (0·9, 1·2)
Central Asia	-5·0 (-5·3, -4·7)	6·1 (5·2, 7·0)	11·2 (9·5, 12·9)	5·9 (5·2, 6·7)	3·1 (2·7, 3·4)	12·7 (11·3, 14·2)	60·4 (21·9, 77·3)	4·7 (3·7, 5·7)
Eastern Europe	-8·8 (-9·3, -8·2)	99·2 (95·5, 100·0)	20·7 (-8·9, 39·7)	10·4 (9·3, 11·5)	5·8 (4·9, 6·8)	46·9 (17·4, 67)	82·4 (67·9, 85·6)	55·8 (50·5, 60·8)
Central Europe	-9·8 (-10·1, -9·6)	7·9 (7·7, 8·1)	24·5 (-4·3, 49·1)	6·0 (5·4, 6·6)	13·6 (11·9, 15·3)	22·2 (20·2, 24·3)	63·8 (61·6, 65·8)	16·3 (14·9, 17·6)
Western Europe	-7·3 (-7·6, -7·0)	34·5 (27·5, 42·1)	28·6 (1·2, 31·8)	4·8 (4·1, 5·3)	21·0 (18·8, 23·8)	49·5 (-15·5, 77)	62·3 (51·2, 66·7)	93·1 (62·4, 99·7)
Oceania	-2·7 (-4·8, -0·1)	1·7 (1·1, 2·3)	13·2 (9·2, 24·5)	4·8 (4·2, 17·1)	-1·1 (-5·8, 6·7)	-4·6 (-4·9, -4·3)	35·2 (16·6, 67·8)	2·2 (1·9, 2·6)
Australasia	-4·1 (-4·3, -4·0)	54·6 (-70·0, 98·3)	11·9 (-8·4, 41·6)	2·9 (2·4, 3·6)	-3·2 (-7·6, 0·9)	-33·4 (-61·9, 3·6)	69·1 (46·6, 73·2)	94·6 (69·6, 99·8)

**Fig 4:**
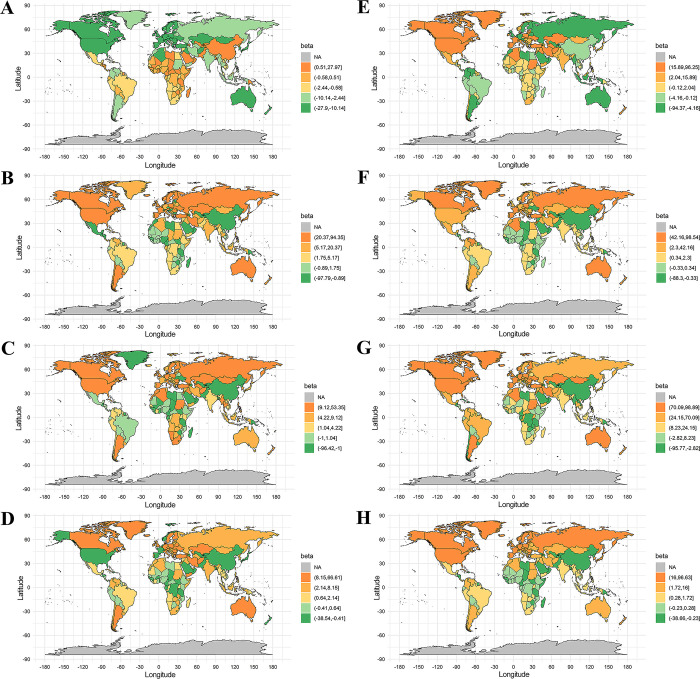
Ecological trend analyses for the SEVs (%) of selected risk factors with ASIR (1/100,000) of IHD at the country level. High body mass index (A), Non-exclusive breastfeeding (B), Diet high in trans-fatty acids (C), Diet low in calcium (D), Occupational ergonomic factors (E), Household air pollution from solid fuels (F), Occupational particulate matter, gases, and fumes (G), Vitamin A deficiency (H). Note: the base layer of the maps were profiled by using R package of “maps”, which referred to the website of https://gadm.org/index.html.

**Fig 5:**
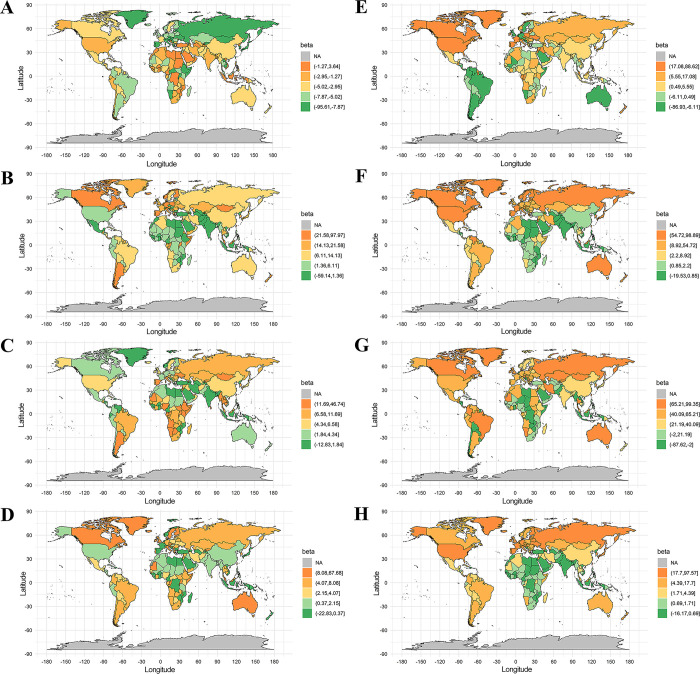
Ecological trend analysis for the SEVs (%) of selected risk factors with ASIR (1/100,000) of all type stroke at the country level. High body mass index (A), Non-exclusive breastfeeding (B), Diet high in trans-fatty acids (C), Diet low in calcium (D), Occupational ergonomic factors (E), Household air pollution from solid fuels (F), Occupational particulate matter, gases, and fumes (G), Vitamin A deficiency (H). Note: the base layer of the maps were profiled by using R package of “maps”, which referred to the website of https://gadm.org/index.html.

Ecological trend analyses at the country level demonstrated a significant positive association of high body mass index with IHD but an inverse association with stroke in 34 countries including China, Pakistan and Uzbekistan, as shown in **Figs 4 and 5** and **S5 Table in S1 File**. The association pattern of inversely with IHD but positively with stroke was observed for diet high in trans-fatty acids in 17 countries, non-exclusive breastfeeding in 18 countries, diet low in calcium in 16 countries, household air pollution from solid fuels in 36 countries, occupational particulate matter, gases, and fumes in 11 countries, and Vitamin A deficiency in 34 countries. The reverse association pattern with IHD and stroke was also found for occupational ergonomic factors, with a positive association with IHD but an inverse association with stroke in 11 countries, and an inverse association with IHD but a positive association with stroke in 14 countries.

Further mutual adjustment showed positive associations of diet high in trans-fatty acids and household air pollution from solid fuels with IHD and stroke, and inverse associations of diet low in calcium and occupational ergonomic factors with the diseases. The associations of high body mass index, non-exclusive breastfeeding, occupational particulate matter, gases and fumes, and Vitamin A deficiency with IHD were also reverse to those with stroke, with mutually adjusted β(95%CI) of -0.29 (-0.65, 0.07), -0.06 (-0.27, 0.15), 2.20 (0.02, 4.38) and 0.41 (0.22, 0.61), respectively, with IHD, and 0.59 (0.38, 0.79), 0.70 (0.58, 0.81), -0.72 (-1.91, 0.47), and -0.29 (-0.40, -0.18), respectively, with stroke (**S5 Table in S1 File**).

Stratified analyses demonstrated reverse associations of the eight factors with IHD and stroke in male and female populations, but consistent associations across countries classified by the World Bank income levels, as shown in **S6 Table in S1 File**.

## Discussion

This ecological study provides updated incidences and temporal trends of IHD and stroke, and explores potential factors driving the discrepant trends at the population level. The three main findings in this analysis included: 1) while the ASIR of stroke decreased globally and regionally during 1990 and 2019, the ASIR of IHD decreased globally, but increased in Eastern Sub-Saharan Africa, Western Sub-Saharan Africa, East Asia, Central Asia and Oceania, particularly in Uzbekistan, Tajikistan, Pakistan, Ukraine, Guam, China, and other 50 countries; 2) the SEVs and the temporal trends of the eight selected risk factors greatly varied in male and female populations, and across the countries with high, upper-middle, lower-middle or low income levels; 3) the SEVs of eight selected risk factors were reversely related with the ASIR of IHD and stroke in 1990, 2000, 2010 and 2019, as well as over the period of 1990 and 2019 in Western Sub-Saharan Africa, East Asia, and Oceania, but consistently associated with the ASIR of IHD and stroke in countries classified by the World Bank income levels. The findings indicate the contribution of the altered SEVs of the risk factors in the discrepant trends of IHD and stroke, and the determinant role of socioeconomic development in the changing patterns of the risk factors and the incidences of IHD and stroke.

The global incidences of IHD and stroke declined over past decades, which have been well-recognized previously [12]. The tremendous decline in incidences of IHD and stroke in high-income countries has been attributable to better health care services and healthier lifestyle in the populations [4]. Starkly contrasting to the declining trends globally, a significant increase in IHD incidence was observed in countries experiencing a rapid socioeconomic translation like Uzbekistan, Tajikistan, Azerbaijan, and China, indicating the influences of the shifts in population risk profiles and the changes in local health policies and accessibility to healthcare [33]. Similarly the rise in stroke incidence in countries like Zimbabwe [34] and Kiribati [35] suggests the potential impacts of limited healthcare resources, delayed implementation of effective prevention strategies, and increase in essential risk factors such as hypertension and smoking [36]. Stratified analysis also showed higher incidences of IHD and stroke in middle-income countries. The variation in disease trends across different income-level regions not only reveal the multifaceted nature of global health, but also highlight the influence of socioeconomic factors on disease burdens.

Although the ASIR of IHD and stroke were observed to decrease in both men and women and in the elderly and the younger age-groups, the incidences were higher in men than women, and in the elderly than the younger people. The sex differences in incidences can be explained by the different levels of risk exposures such as occupational particulate matter, gases, and fumes and body fat distribution observed in this and previous studies [37], the sex hormones, and the sex-specific molecular mechanisms that affect glucose and lipid metabolism, and ultimately cardiac energy metabolisms and functions [38, 39]. The higher incidence in the elderly has been attributed to the reduction in elasticity of blood vessels and insufficient compensatory capacity in the subgroup than in younger populations [40–42]. Considering that age and gender were inherent factors, interventions should be developed targeting modifiable factors to alleviate the burden of cardiovascular diseases.

IHD and stroke shared multiple common risk factors, including smoking, overweight/obesity, high blood pressure, diabetes, and abnormal serum lipid [22]. The biological mechanisms underlying are also illuminated in recent years. For example, overwhelming evidence proved the important role of high cholesterol and high blood pressure levels in the development of CVD [23]. Smoking was associated with an elevated risk of CVD by leading to insulin resistance, hypercholesterolemia, and elevated catecholamine levels that cause higher heart rate and blood pressure [24, 25]. The changes in distributions of the common risk factors may have led to similar temporal trends in incidence of IHD and stroke, which were observed in most regions and countries, but not in some regions and countries. This might be explained by the different structures of the cardiovascular and cerebrovascular systems. Cardiovascular vessels have an internal elastic lamina, whereas intracranial arteries just have thin intima without external elastic membrane. In addition, elastic fibers in the middle and outer membranes of intracranial arteries are fewer than those of extracranial arteries with a same diameter [43], making cerebrovascular vessels prone to rupture and bleeding. However, the human brain has adequate blood supply and compensatory mechanisms, while IHD would be triggered by cardiomyocytes ischemia and hypoxia due to coronary artery stenosis [44].

An alternative explanation for the contrasting incidence trends of IHD and stroke is the altered exposures to specific risk factors for IHD and stroke. Evidence is accumulating on respective risk factors for IHD and stroke at the individual level [26]. Circulating calcium, the risk factor for vascular disease, was found to predict myocardial infarction independently [29]. Higher dietary calcium intake was linked to a lower risk of hypertension [30], whereas among the similar risk factor profiles among East Asian and European ancestry populations, only genetically predicted elevated blood pressure was significantly associated with IS in East Asians [45]. Plasma retinol level was found to be associated with a lower risk of first stroke among Chinese hypertensive adults [46]. In a cohort study of 320,249 participants, being breastfed was related to lower cardiovascular risk factors, and associated with reduced risks of CVD events and deaths, particularly for the myocardial infarction events and deaths [47]. In a recent study using the China Kadoorie Biobank data, sex-specific models were developed separately for IHD, IS, and HS based on the respective hazard ratios for the predictors with the subtype of CVD [48]. In this study, we observed significant reverse associations of the eight risk factors with the incidences of IHD and stroke at the population level in Western Sub-Saharan Africa, East Asia, and Oceania, and in 59 countries, which indicate the contributions of altered risk exposures specific for IHD and stroke in the discrepant secular trends of the two major subtypes of CVD. The alternation in risk exposures underlying the temporal trends, once confirmed, will deepen our understanding of the mechanisms of CVD, and help to develop targeted interventions to curb the upward trends of the diseases.

It is of note that the exposures to almost all of the selected risk factors were determined by socioeconomic development [27]. High body-mass index, non-exclusive breastfeeding, and diet high in trans-fatty acids were more common in high-income countries [49–51], while diet low in calcium, Vitamin A deficiency, household air pollution from solid fuels, occupational particulate matter, gases and fumes, and occupational ergonomic factors were related to low socio-economic levels [52–54]. In this study, we observed higher but decreased diet high in trans-fatty acids in high-income countries, and lower but increased high body-mass index in countries with lower income levels, supporting the effects of altered risk factor distribution due to socioeconomic development on the risks of IHD and stroke. In addition, the associations of the SEVs of selected risk factors with IHD and stoke were consistent in sub-group of countries classified by the World Bank income level, indicating the determinant role of socioeconomic levels in the secular trends of incidences of IHD and stroke. For example, the negative associations of high body-mass index with ASIR of IHD and stroke at country level were observed consistently positive across the countries classified by the World Bank income level, suggesting the strong confounding effect of socioeconomic development.

Rapid economic transitions are usually accompanied with drastic lifestyle changes, such as shifts in dietary patterns and deteriorating work environments, which increased exposures to specific cardiovascular risk factors. The complex interplay between socioeconomic development and public health outcomes necessitated targeted interventions to address unique regional challenges. For instance, the contrasting trends in incidence of IHD between North America and East Asia highlight the significant impact of specific risk factors driven by socioeconomic transformations and public health policies. In North America, public health initiatives and improvements in healthcare accessibility helped reduce the consumption of diets high in trans-fatty acids [55], one of important risk factors for IHD, and contributed to a mitigation of the disease burden in recent years [56]. Meanwhile, the rapid urbanization and economic development in East Asian greatly elevated body mass index and increased the consumption of diets high in trans-fatty acids [57], leading to a rising burden of IHD [58]. Our analysis emphasized the importance of addressing regional specific exposures that reflect unique health challenges. There was a critical demand to delve into the temporal distribution of the changes that previous studies had not focused on, driven by the urgent requirement for localized health data to inform effective public health strategies.

## Strengths and limitations

This is the first study exploring the potential factors underlying the different temporal trends in ASIR of IHD and stroke in ecological perspective. The GBD data provides global, regional and national incidence of IHD, all subtypes of stroke during 1990 and 2019, enabling us to describe their incidences and secular trends. Moreover, we identified potential factors contributing to the discrepant temporal trends of IHD and stroke according to the directions of correlations and associations of various risk factors with the diseases, which was based on the hypothesis that the discrepant trends in ASIR of IHD and stroke are determined by the distributions of their specific risk factors. The novel method makes the selection of the potential risk factors more efficiently and accurately.

Several limitations exist in this study. First, as an ecological study, the results of this analysis were only used to guide the generation of hypotheses, but not to make a causal inference. Despite the critical limitation, an ecological study is the only design that could explore the drivers underlying the increasing trend of IHD but the decreased incidence of stroke in some regions or countries. Moreover, as the ecological trend analysis is less prone to ecological fallacy, the evidence derived from this study was relatively reliable. Second, we only evaluated the risk factors available in the GBD 2019, and may have missed other important risk factors specific for IHD and stroke. As an exploratory study, however, this analysis aimed to examine the existence of specific risk factors for IHD and stroke, but not to identify all potential risk factors for CVD. Finally, the increased incidences of IHD in regions or countries experiencing a rapid socioeconomic translation may be due to improved diagnoses and registry systems. In these regions or countries, nevertheless, the ASIR of stroke was observed to decrease during the same period, mitigating our concern on the influence of elevated completeness of registry data on the upward trend of IHD. Moreover, the SEVs of risk factors, which increased or decreased during the period of 1990 and 2019, may have also affected by the improved quality of registry data. The simultaneously improved data on risk factors and incidences might not greatly bias our evaluation for their associations.

## Conclusions

This study describes the incidences and secular trends of IHD and stroke at the global, regional, and national levels, demonstrates higher ASIR of IHD (333.5 in men and 198.5 in women per 100,000) than stroke (178.5 in men and 182.0 in women per 100,000) in 2019, and highlights the contributing factors underlying the discrepant trends in 3 of 21 regions and 56 of 204 countries experiencing rapid socioeconomic translation. Our findings indicate the influences of altered exposures to diet high in trans-fatty acids, high body mass index, non-exclusive breastfeeding, diet low in calcium, household air pollution from solid fuels, occupational ergonomic factors, Vitamin A deficiency, and occupational particulate matter, gases, and fumes across countries, and the potential determinant of socioeconomic development of the countries, which bring us insight into the respective mechanisms and targeted intervention strategies for the cardiovascular and cerebrovascular diseases.

## Supporting information

S1 File (DOCX)
