# Supplementary material for: Incidence trends and specific risk factors of ischemic heart disease and stroke: An ecological analysis based on the Global Burden of Disease 2019
Source: PLOS Glob Public Health. 2024 Nov 20;4(11):e0003920. doi: 10.1371/journal.pgph.0003920 (PMC11578511; doi:10.1371/journal.pgph.0003920)
Supplement: S1 File — (DOCX) [file pgph.0003920.s001.docx]

**S1 Table**: Age-standardized incidence (1/100000) of ischemic heart disease and all type stroke in 2019 and AAPC in 1990-2019 in men and women

|  | Men | | | | | |  | | Women | | | | | | | | | | | | |
| --- | --- | --- | --- | --- | --- | --- | --- | --- | --- | --- | --- | --- | --- | --- | --- | --- | --- | --- | --- | --- | --- |
|  | ischemic heart disease | |  | all type of stroke | | |  | | ischemic heart disease | | | |  | | | all type of stroke | | | | |  |
|  | ASIR (95%UI) | AAPC (95%CI) |  | ASIR(95%UI) | AAPC (95%CI) |  | | ASIR (95%UI) | | AAPC (95%CI) | |  | | | ASIR (95%UI) | | | AAPC (95%CI) | |  |  |
| Global | 333.5 (297.0,371.9) | -0.70 (-0.82,-0.57) |  | 151.1 (136.9,167.5) | -0.58 (-0.62,-0.55) |  | | 198.5 (176.4,221.2) | | | -0.68 (-0.78,-0.58) | | |  | | | 149.8 (135.6,166.6) | | -0.68 (-0.71,-0.65) | |  |
| By region |  |  |  |  |  |  | |  | | |  | | |  | | |  | |  | |  |
| Eastern Sub-Saharan Africa | 250.2 (219.2, 284.3) | 0.11 (0.08, 0.15) |  | 160.2 (147.1, 176) | -0.79 (-0.82,-0.77) |  | | 162.0 (140.6, 184.7) | | | 0.08 (0.06,0.09) | | |  | | | 155.6 (142.8, 170.3) | | -0.81 (-0.83,-0.78) | |  |
| Western Sub-Saharan Africa | 261.8 (228.5, 297.6) | 0.19 (0.18, 0.20) |  | 145.6 (134.5, 158.9) | -0.34 (-0.35,-0.32) |  | | 181.0 (156.8, 206.1) | | | 0.20 (0.12,0.28) | | |  | | | 170.9 (157.2, 186.2) | | -0.45 (-0.47,-0.43) | |  |
| Southern Sub-Saharan Africa | 303.7 (265.2, 346.9) | -0.03 (-0.08, 0.03) |  | 158.1 (140, 180.1) | -0.27 (-0.30,-0.23) |  | | 202.2 (175.5, 230.7) | | | -0.26 (-0.29,-0.22) | | |  | | | 165.9 (147.4, 189) | | 0.06 (-0.01,0.13) | |  |
| Central Sub-Saharan Africa | 255.7 (223.9, 290.1) | -0.16 (-0.18, -0.15) |  | 161.4 (148.6, 176.4) | -0.66 (-0.68,-0.65) |  | | 203.8 (180.0, 230.0) | | | -0.10 (-0.20,-0.01) | | |  | | | 162.6 (149.3, 178.4) | | -0.61 (-0.63,-0.60) | |  |
| North Africa and Middle East | 708.1 (639.6, 780.7) | -0.40 (-0.43,-0.36) |  | 171.7 (156.3, 189.5) | -0.18 (-0.19,-0.18) |  | | 514.5 (465.3, 567.0) | | | -0.27 (-0.30,-0.24) | | |  | | | 194.7 (177.5, 214) | | -0.20 (-0.22,-0.19) | |  |
| Caribbean | 427.3 (376.0, 481.8) | -0.16 (-0.31,-0.01) |  | 126.9 (117.3, 137.4) | -0.27 (-0.33,-0.21) |  | | 311.5 (273.6, 353.1) | | | -0.24 (-0.26,-0.22) | | |  | | | 131.6 (121.8, 142.7) | | -0.36 (-0.37,-0.34) | |  |
| Central Latin America | 232.3 (204.6, 262.8) | -0.22 (-0.26,-0.17) |  | 88.1 (80.4, 96.7) | -1.12 (-1.20,-1.04) |  | | 148.5 (130.2, 168.1) | | | -0.48 (-0.58,-0.39) | | |  | | | 94.7 (86.4, 104.3) | | -1.43 (-1.47,-1.39) | |  |
| Southern Latin America | 237.5 (210.2, 267.9) | -1.24 (-1.40, -1.08) |  | 91.2 (84.3, 98.5) | -1.53 (-1.59,-1.47) |  | | 100.2 (88.6, 113.3) | | | -1.54 (-1.68,-1.40) | | |  | | | 88.6 (81.3, 96.9) | | -1.43 (-1.47,-1.40) | |  |
| Andean Latin America | 106.2 (92.7, 119.7) | -0.34 (-0.55, -0.13) |  | 86.2 (79.2, 94) | -0.83 (-0.87,-0.79) |  | | 53.2 (46.8, 60.6) | | | -0.32 (-0.69,0.05) | | |  | | | 89.4 (82.3, 97.2) | | -1.03 (-1.05,-1.01) | |  |
| Tropical Latin America | 147.6 (130.1, 165.9) | -0.47 (-0.71, -0.23) |  | 138.6 (123.5, 157.5) | -1.88 (-2.03,-1.73) |  | | 78.0 (68.8, 87.9) | | | -0.48 (-0.71,-0.26) | | |  | | | 118.2 (106.7, 130.8) | | -1.95 (-1.99,-1.90) | |  |
| High-income North America | 233.1 (213.2, 255.7) | -2.81 (-2.88, -2.75) |  | 74 (66.1, 83.3) | -1.22 (-1.32,-1.12) |  | | 129.8 (118.2, 142.7) | | | -2.50 (-2.60,-2.39) | | |  | | | 93.6 (82.9, 105.5) | | -1.00 (-1.07,-0.93) | |  |
| High-income Asia Pacific | 169.5 (147.6, 192.2) | -0.86 (-0.93, -0.79) |  | 126.5 (114.6, 139.9) | -1.39 (-1.42,-1.36) |  | | 78.5 (67.9, 89.6) | | | -1.03 (-1.08,-0.97) | | |  | | | 137.3 (123.1, 152.9) | | -1.00 (-1.03,-0.97) | |  |
| East Asia | 235.3 (209.4, 264.3) | 0.30 (0.18, 0.42) |  | 209 (185.6, 238.2) | -0.32 (-0.43,-0.21) |  | | 163.5 (145.1, 184.7) | | | 0.40 (0.36,0.44) | | |  | | | 194.2 (169.8, 224.7) | | -0.40 (-0.49,-0.30) | |  |
| South Asia | 559.7 (491.2, 630.3) | 0.04 (-0.16, 0.23) |  | 114.9 (103.8, 128.1) | -0.40 (-0.42,-0.37) |  | | 299.6 (261.9, 338.6) | | | 0.06 (-0.05,0.16) | | |  | | | 119.6 (108, 133.1) | | -0.35 (-0.40,-0.29) | |  |
| Southeast Asia | 182.1 (161.7, 202.8) | -0.33 (-0.50, -0.16) |  | 230.8 (211.7, 253.8) | -0.20 (-0.22,-0.18) |  | | 96.1 (85.3, 107.3) | | | -0.30 (-0.60,-0.01) | | |  | | | 201.3 (183.4, 222.9) | | -0.32 (-0.33,-0.31) | |  |
| Central Asia | 829.5 (759.3, 904.6) | 0.17 (-0.03, 0.37) |  | 212.2 (199.3, 227.1) | -0.46 (-0.50,-0.41) |  | | 514.4 (470.5, 560.1) | | | 0.28 (0.18,0.38) | | |  | | | 180.6 (168.2, 194.2) | | -0.73 (-0.77,-0.69) | |  |
| Eastern Europe | 631.5 (556.2, 711.8) | -0.29 (-0.41, -0.18) |  | 200.6 (178.8, 225.1) | -0.86 (-0.91,-0.80) |  | | 426.2 (373.9, 482.7) | | | -0.16 (-0.22,-0.09) | | |  | | | 181.3 (161.3, 204.8) | | -1.20 (-1.22,-1.17) | |  |
| Central Europe | 327.3 (296.2, 359.3) | -1.61 (-1.97, -1.24) |  | 154.8 (141.2, 169.5) | -1.33 (-1.35,-1.31) |  | | 191.8 (173.9, 210.5) | | | -1.49 (-1.91,-1.06) | | |  | | | 145.8 (132, 161.3) | | -1.55 (-1.58,-1.52) | |  |
| Western Europe | 296.2 (264.7, 329.3) | -1.61 (-1.64, -1.57) |  | 69.4 (63.5, 76.4) | -1.96 (-2.01,-1.91) |  | | 123.6 (110.5, 137.0) | | | -1.84 (-1.92,-1.77) | | |  | | | 69.5 (62.8, 76.4) | | -1.80 (-1.87,-1.73) | |  |
| Oceania | 258.2 (225.9, 292.2) | 0.07 (-0.05, 0.19) |  | 218.5 (203.9, 234.5) | -0.34 (-0.35,-0.33) |  | | 157.0 (136.3, 180.2) | | | 0.08 (0.04, 0.13) | | |  | | | 214.2 (199.9, 230.7) | | -0.15 (-0.16,-0.14) | |  |
| Australasia | 528.7 (463.5, 599.0) | -1.15 (-1.22, -1.08) |  | 60.5 (54.7, 66.6) | -2.05 (-2.09,-2.01) |  | | 177.5 (156.6, 199.2) | | | -1.33 (-1.40,-1.27) | | |  | | | 69.1 (62.1, 76.8) | | -1.52 (-1.58,-1.46) | |  |
| Countries by World bank income level | |  |  |  |  |  | |  | | |  | | |  | | |  | |  | |  |
| High income | 265.9 (238.2,294.8) | -1.84 (-1.89,-1.78) |  | 88.6 (80.7,97.3) | -1.49 (-1.53,-1.45) |  | | 125.8 (112.6,139.4) | | | -1.92 (-2.04,-1.80) | | |  | | | 95.4 (86.5,105.3) | | -1.28 (-1.35,-1.21) | |  |
| Upper middle income | 280.7 (250.2,313.6) | -0.43 (-0.52,-0.34) |  | 184.9 (165.5,208.7) | -0.54 (-0.63,-0.46) |  | | 199.1 (176.8,223.1) | | | -0.49 (-0.54,-0.44) | | |  | | | 174.7 (155.0,198.4) | | -0.75 (-0.78,-0.71) | |  |
| Lower middle income | 490.3 (435.1,548.1) | -0.03 (-0.20,0.14) |  | 153.8 (140.6,169.9) | -0.34 (-0.36,-0.33) |  | | 284.1 (251.5,317.6) | | | -0.22 (-0.36,-0.07) | | |  | | | 155.1 (141.3,170.8) | | -0.50 (-0.53,-0.46) | |  |
| Low income | 297.5 (262.2,333.5) | -0.05 (-0.06,-0.04) |  | 167.1 (154.8,181.7) | -0.50 (-0.53,-0.47) |  | | 198.5 (175.5,223.2) | | | -0.01 (-0.07,0.06) | | |  | | | 174.1 (161.1,188.4) | | -0.44 (-0.46,-0.42) | |  |

*ASIR: Age-standardized incidence rate; AAPC: Average annual percent change; UI: Uncertainty Interval; CI: Confidence Interval

**S2 Table**: Age-standardized incidence (1/100000) and AAPC (%) of three subtypes of stroke

|  | Ischemic stroke | |  | Intracerebral hemorrhage | |  | Subarachnoid hemorrhage | |
| --- | --- | --- | --- | --- | --- | --- | --- | --- |
|  | ASIR (95%UI) in 2019 | AAPC (95%CI) |  | ASIR (95%UI) in 2019 | AAPC (95%CI) |  | ASIR (95%UI) in 2019 | AAPC (95%CI) |
| **Global** | 94.5 (81.9,110.8) | -0.36 (-0.38,-0.34) |  | 41.8 (36.5,47.9) | -1.20 (-1.31,-1.09) |  | 14.5 (12.3,16.9) | -0.63 (-0.71,-0.56) |
| **By sex** |  |  |  |  |  |  |  |  |
| Male | 90.9 (78.5,106.6) | -0.23 (-0.26,-0.20) |  | 47.2 (41.4,53.9) | -1.15 (-1.25,-1.05) |  | 13.0 (11.1,15.3) | -0.63 (-0.70,-0.57) |
| Female | 97.2 (84.1,113.9) | -0.42 (-0.45,-0.40) |  | 36.8 (32.2,42.2) | -1.27 (-1.34,-1.19) |  | 15.7 (13.4,18.3) | -0.64 (-0.70,-0.59) |
| **By region** |  |  |  |  |  |  |  |  |
| Eastern Sub-Saharan Africa | 85.6 (74.5, 98.9) | -0.13(-0.15, -0.12) |  | 62.6 (56.5, 69.7) | -1.56(-1.61, -1.51) |  | 9.6 (7.8, 11.6) | -0.59(-0.62, -0.55) |
| Western Sub-Saharan Africa | 77.8 (68.0, 89.3) | -0.09(-0.11, -0.07) |  | 73.7 (66.0, 82.4) | -0.68(-0.71, -0.65) |  | 7.5 (6.1, 9.1) | -0.42(-0.44, -0.41) |
| Southern Sub-Saharan Africa | 113.0 (96.1, 134.8) | 0.23(0.20, 0.27) |  | 43.5 (37.5, 50.5) | -0.74(-0.80, -0.68) |  | 7.1 (5.9, 8.6) | -0.11(-0.16, -0.07) |
| Central Sub-Saharan Africa | 84.2 (72.9, 96.6) | -0.29(-0.33, -0.25) |  | 68.1 (62.4, 75.0) | -1.05(-1.09, -1.02) |  | 10.0 (8.2, 12.2) | -0.18(-0.19, -0.17) |
| North Africa and Middle East | 135.5 (119.7, 153.6) | 0.29(0.28, 0.31) |  | 35.0 (31.8, 38.6) | -1.35(-1.39, -1.32) |  | 12.5 (10.7, 14.7) | -1.05(-1.10, -1.00) |
| Caribbean | 77.3 (68.4, 87.2) | -0.18(-0.21, -0.15) |  | 39.8 (36.9, 43.1) | -0.62(-0.65, -0.58) |  | 12.4 (10.8, 14.3) | -0.15(-0.18, -0.13) |
| Central Latin America | 55.6 (48.3, 64.3) | -1.40(-1.46, -1.34) |  | 21.2 (18.9, 23.9) | -1.64(-1.68, -1.60) |  | 14.7 (12.8, 17.2) | -0.13(-0.15, -0.10) |
| Southern Latin America | 51.6 (45.1, 58.8) | -1.23(-1.28, -1.17) |  | 22.7 (20.9, 24.9) | -2.16(-2.22, -2.10) |  | 15.6 (13.8, 17.8) | -1.23(-1.26, -1.20) |
| Andean Latin America | 50.5 (44.3, 57.6) | -0.61(-0.63, -0.59) |  | 21.1 (19.1, 23.4) | -1.72(-1.77, -1.68) |  | 16.3 (14.4, 18.6) | -0.71(-0.73, -0.68) |
| Tropical Latin America | 78.3 (66.4, 93.0) | -1.89(-1.95, -1.83) |  | 31.7 (26.8, 37.4) | -2.49(-2.57, -2.42) |  | 17.2 (14.5, 20.6) | -0.86(-0.89, -0.82) |
| High-income North America | 56.8 (47.8, 67.1) | -1.36(-1.41, -1.19) |  | 13.4 (11.3, 15.9) | -0.71(-0.76, -0.66) |  | 14.6 (12.3, 17.5) | -0.38(-0.42, -0.34) |
| High-income Asia Pacific | 63.3 (54.0, 74.4) | -1.94(-2.00, -1.89) |  | 18.3 (16.0, 21.0) | -2.65(-2.77, -2.53) |  | 51.4 (43.1, 60.4) | 1.20(1.02, 1.38) |
| East Asia | 143.6 (120.8, 171.8) | 0.98(0.97, 0.99) |  | 45.5 (38.6, 53.4) | -2.56(-2.78, -2.34) |  | 11.5 (9.8, 13.5) | -1.67(-1.84, -1.50) |
| South Asia | 50.5 (43.0, 58.9) | -0.15(-0.18, -0.13) |  | 55.8 (47.5, 65.6) | -0.49(-0.52, -0.46) |  | 11.1 (9.4, 13.2) | -0.73(-0.79, -0.67) |
| Southeast Asia | 115.9 (99.6, 135.9) | 0.15(0.10, 0.19) |  | 85.8 (76.4, 96.9) | -0.68(-0.70, -0.66) |  | 14.2 (12.3, 16.6) | -0.55(-0.57, -0.53) |
| Central Asia | 103.0 (91.5, 116.0) | -0.53(-0.56, -0.51) |  | 75.7 (71.1, 81.4) | -0.77(-0.86, -0.69) |  | 16.5 (14.4, 18.9) | -0.23(-0.27, -0.19) |
| Eastern Europe | 134.6 (114.7, 158.1) | -1.16(-1.19, -1.13) |  | 33.2 (28.6, 38.6) | -1.22(-1.28, -1.17) |  | 24.2 (20.5, 28.6) | -0.16(-0.21, -0.11) |
| Central Europe | 109.8 (96.5, 125.2) | -1.28(-1.31, -1.25) |  | 25.6 (23.6, 27.9) | -2.4(-2.48, -2.32) |  | 15.3 (13.3, 17.7) | -0.85(-0.87, -0.83) |
| Western Europe | 46.3 (40.3, 52.9) | -2.15(-2.20, -2.09) |  | 11.8 (10.5, 13.2) | -1.96(-2.05, -1.88) |  | 11.7 (10.0, 13.9) | -0.42(-0.45, -0.39) |
| Oceania | 80.7 (69.9, 93.3) | -0.06(-0.08, -0.03) |  | 113.7 (105.0, 123.3) | -0.34(-0.35, -0.32) |  | 22.1 (19.4, 25.1) | -0.38(-0.40, -0.37) |
| Australasia | 44.3 (38.3, 50.5) | -2.01(-2.06, -1.97) |  | 9.6 (8.6, 10.7) | -1.71(-1.81, -1.61) |  | 11.2 (9.6, 13.1) | -0.59(-0.68, -0.50) |

*ASIR: Age-standardized incidence rate; AAPC: Average annual percent change; UI: Uncertainty Interval; CI: Confidence Interval

**S3 Table:** Global summary exposure values (SEVs) of selected risk factors in male and female populations

| Selected factors | SEV (95%UI) in 2019 | AAPC (95%CI) | Men | |  | Women | |
| --- | --- | --- | --- | --- | --- | --- | --- |
|  |  |  | SEV (95%UI) in 2019 | AAPC (95%CI) |  | SEV (95%UI) in 2019 | AAPC (95%CI) |
| Diet high in trans-fatty acids | 44.7 (37.6, 58.8) | -0.43 (-0.45, -0.41) | 44.3(37.4,58.8) | -0.44 (-0.46,-0.42) |  | 45.0(37.5,60.2) | -0.41 (-0.43,-0.39) |
| Diet low in calcium | 46.0 (35.9, 60.3) | -0.46 (-0.48, -0.45) | 48.5(38.2,62.7) | -0.49 (-0.50,-0.48) |  | 43.7(33.4,58.1) | -0.44 (-0.45,-0.43) |
| High body-mass index | 19.5 (15.6, 24.4) | 1.96 (1.93, 1.98) | 18.3(14.2,23.4) | 2.22 (2.20,2.23) |  | 20.6(16.7,25.8) | 1.75 (1.73,1.78) |
| Household air pollution from solid fuels | 11.7 (6.6, 18.3) | -2.83 (-2.97, -2.69) | 11.1(6.1,17.6) | -2.96 (-3.10,-2.82) |  | 12.3(7.1,19.1) | -2.71 (-2.85,-2.57) |
| Non-exclusive breastfeeding | 18.4 (12.9, 25.5) | -0.53 (-0.59, -0.47) | 18.4(12.9,25.5) | -0.53 (-0.59,-0.47) |  | 18.4(12.9,25.6) | -0.52 (-0.58,-0.47) |
| Occupational ergonomic factors | 17.5 (16.2, 19.2) | -0.71 (-0.74, -0.68) | 21.5(19.8,23.6) | -0.73 (-0.76,-0.70) |  | 13.5(12.2,14.8) | -0.68 (-0.71,-0.65) |
| Vitamin A deficiency | 15.0 (13.6, 16.9) | -2.75 (-2.91, -2.58) | 12.7(10.0,16.5) | -3.00 (-3.16,-2.84) |  | 7.5(5.9,9.9) | -2.40 (-2.56,-2.24) |
| Occupational particulate matter, gases, and fumes | 10.1 (8.4, 12.1) | -0.09 (-0.10, -0.09) | 16.3(13.6,19.4) | -0.16 (-0.16,-0.15) |  | 13.6(12.1,15.4) | 0.01 (0.00,0.01) |

* SEV: summary exposure values; AAPC: Average annual percent change; UI: Uncertainty Interval; CI: Confidence Interval

**S4 Table:** Global summary exposure values (SEVs) of selected risk factors by the World Bank income levels

| Selected factors | High-income countries | |  | Upper-middle income countries | |  | Lower-middle income countries | |  | Low-income countries | |
| --- | --- | --- | --- | --- | --- | --- | --- | --- | --- | --- | --- |
|  | SEV (95%UI) in 2019 | AAPC  (95%CI) |  | SEV (95%UI) in 2019 | AAPC  (95%CI) |  | SEV (95%UI) in 2019 | AAPC  (95%CI) |  | SEV (95%UI)  in 2019 | AAPC (95%CI) |
| Diet high in trans-fatty acids | 53.4  (47.1, 65.2) | -0.30  (-0.32, -0.28) |  | 39.1  (31.9, 55.9) | -0.32  (-0.33, -0.31) |  | 48.7  (41.0, 62.9) | -0.40  (-0.42, -0.37) |  | 30.5  (22.7, 50.9) | -0.44  (-0.46, -0.42) |
| Diet low in calcium | 18.2  (10.3, 31.1) | -0.38  (-0.41 -0.34) |  | 42.4  (30.6, 60.4) | -0.19  (-0.20, -0.18) |  | 59.5  (49.3, 73.1) | -0.48  (-0.50, -0.47) |  | 79.4  (70.8, 90.2) | -1.07  (-1.09, -1.06) |
| High body-mass index | 30.8  (24.8, 37.9) | 1.60  (1.59, 1.61) |  | 21.5  (16.7, 27.5) | 2.12  (2.08, 2.15) |  | 15.3  (12.6, 18.6) | 2.75  (2.70, 2.80) |  | 12.3  (9.5, 15.9) | 2.41  (2.39, 2.44) |
| Household air pollution from solid fuels | 0.1  (0.0, 0.3) | -7.33  (-7.40, -7.26) |  | 3.4  (1.6, 6.2) | -1.16  (-1.20, -1.13) |  | 14.5  (8.2, 23.3) | -3.47  (-3.53, -3.41) |  | 46.7  (28.3, 64.5) | -6.34  (-6.46, -6.22) |
| Non-exclusive breastfeeding | 23.0  (19.5, 27.6) | -0.14  (-0.20, -0.09) |  | 20.5  (15.3, 27.4) | -0.62  (-0.67, -0.57) |  | 17.3  (11.4, 24.9) | -0.37  (-0.41, -0.32) |  | 16.0  (10.2, 23.9) | -0.54  (-0.57, -0.51) |
| Occupational ergonomic factors | 9.8  (8.5, 11.5) | -0.13  (-0.19, -0.08) |  | 16.4  (14.7, 18.2) | -0.55  (-0.60, -0.50) |  | 19.5  (18.0, 21.2) | -0.50  (-0.56, -0.43) |  | 31.6  (30.1, 33.3) | -1.48  (-1.52, -1.44) |
| Vitamin A deficiency | 1.9  (1.6, 2.2) | -2.70  (-2.84, -2.56) |  | 5.6  (4.4, 6.9) | -1.56  (-1.65, -1.47) |  | 14.8  (11.7, 18.3) | -3.84  (-4.12, -3.56) |  | 37.9  (34.6, 41.2) | -4.25  (-4.38, -4.11) |
| Occupational particulate matter, gases, and fumes | 6.2  (4.9, 7.9) | -0.36  (-0.37, -0.36) |  | 11.9  (10.0, 14.1) | -0.12  (-0.12, -0.11) |  | 9.8  (8.2, 11.8) | -0.10  (-0.10, -0.09) |  | 12.5  (10.4, 14.9) | -0.19  (-0.20, -0.19) |

* SEV: summary exposure values; AAPC: Average annual percent change; UI: Uncertainty Interval; CI: Confidence Interval

**S5 Table:** Ecological trend analysis for selected risk factors with ASIR of IHD and all type stroke during 1990 and 2019 at the country level

| Selected risk factors | Countries with IHD↑ & stroke↓ (n=56) | |  | Countries with IHD↓& stroke↑(n=3)* | |  | Mutually adjusted β(95%CI) | |
| --- | --- | --- | --- | --- | --- | --- | --- | --- |
|  | Positively related with IHD & negatively with stroke | Negatively related with IHD & positively with stroke |  | Positively related with IHD & negatively with stroke | Negatively related with IHD & positively with stroke |  | IHD | Stroke |
| High body mass index | 34 | 0 |  | 0 | 1 |  | -0.29 (-0.65, 0.07) | 0.59 (0.38, 0.79) |
| Diet high in trans-fatty acids | 0 | 17 |  | 1 | 0 |  | 3.51 (3.16, 3.85) | 0.39 (0.20, 0.59) |
| Non-exclusive breastfeeding | 0 | 18 |  | 1 | 0 |  | -0.06 (-0.27, 0.15) | 0.70 (0.58, 0.81) |
| Diet low in calcium | 2 | 16 |  | 1 | 0 |  | -1.91 (-2.15, -1.68) | -0.48 (-0.62, -0.35) |
| Occupational ergonomic factors | 10 | 14 |  | 1 | 0 |  | -1.06 (-1.52, -0.60) | -0.92 (-1.18, -0.66) |
| Household air pollution from solid fuels | 0 | 36 |  | 1 | 0 |  | 1.25 (0.89, 1.62) | 1.75 (1.55, 1.96) |
| Occupational particulate matter, gases, & fumes | 3 | 11 |  | 1 | 0 |  | 2.20 (0.02, 4.38) | -0.72 (-1.91, 0.47) |
| Vitamin A deficiency | 0 | 34 |  | 1 | 0 |  | 0.41 (0.22, 0.61) | -0.29 (-0.40, -0.18) |

↑increasing trend in ASIR;↓decreasing trend in ASIR

* Referring to Bosnia and Herzegovina, Timor-Leste, and Viet Nam.

ASIR: Age-standardized incidence rate; CI: Confidence Interval; IHD: ischemic heart disease.

**S6 Table:** Ecological trend analysis for selected risk factors with ASIR of IHD and stroke at the country level by sex and the World Bank income levels

|  | **IHD** | **All type stroke** | **IS** | **ICH** | **SAH** |
| --- | --- | --- | --- | --- | --- |
| **Men** |  |  |  |  |  |
| Diet high in trans-fatty acids | 3.87(1.77,5.97) | -1.47(-2.09,-0.85) | -0.32(-0.68,0.04) | -1.18(-1.58,-0.79) | 0.04(-0.02,0.09) |
| Diet low in calcium | -1.68(-2.63,-0.72) | 1.01(0.75,1.27) | 0.20(0.04,0.36) | 0.85(0.70,1.01) | -0.04(-0.06,-0.02) |
| High body-mass index | 4.98(2.51,7.46) | -0.92(-1.68,-0.16) | 0.26(-0.17,0.68) | -1.19(-1.67,-0.72) | 0.02(-0.04,0.08) |
| Household air pollution from solid fuels | -2.88(-4.43,-1.34) | 0.99(0.53,1.45) | -0.05(-0.32,0.21) | 1.10(0.83,1.38) | -0.06(-0.10,-0.02) |
| Non-exclusive breastfeeding | 7.55(2.46,12.65) | -2.52(-4.05,-1.00) | -0.02(-0.89,0.85) | -2.52(-3.49,-1.56) | 0.02(-0.11,0.15) |
| Occupational ergonomic factors | -3.29(-6.00,-0.57) | 1.37(0.56,2.17) | -0.08(-0.54,0.38) | 1.55(1.05,2.05) | -0.10(-0.17,-0.03) |
| Vitamin A deficiency | -5.49(-15.25,4.26) | 3.07(0.16,5.97) | 0.15(-1.48,1.77) | 3.39(1.52,5.26) | -0.47(-0.70,-0.24) |
| Occupational particulate matter, gases, and fumes | -3.02(-4.65,-1.39) | 1.53(1.07,1.99) | 0.18(-0.10,0.46) | 1.40(1.13,1.67) | -0.05(-0.09,-0.01) |
| **Women** |  |  |  |  |  |
| Diet high in trans-fatty acids | 2.12(0.40,3.84) | -1.21(-1.84,-0.59) | -0.38(-0.81,0.04) | -0.93(-1.27,-0.60) | 0.10(0.02,0.18) |
| Diet low in calcium | -0.71(-1.45,0.02) | 1.03(0.80,1.27) | 0.34(0.16,0.51) | 0.74(0.63,0.86) | -0.04(-0.08,-0.01) |
| High body-mass index | 4.49(2.67,6.31) | -0.46(-1.17,0.25) | 0.44(-0.02,0.91) | -0.92(-1.30,-0.54) | 0.02(-0.07,0.11) |
| Household air pollution from solid fuels | -1.51(-2.65,-0.36) | 1.08(0.67,1.49) | 0.11(-0.18,0.39) | 1.08(0.89,1.27) | -0.11(-0.17,-0.06) |
| Non-exclusive breastfeeding | 5.00(1.10,8.90) | -2.32(-3.76,-0.87) | -0.31(-1.28,0.66) | -1.98(-2.76,-1.21) | -0.03(-0.21,0.16) |
| Occupational ergonomic factors | -2.90(-4.75,-1.05) | 0.77(0.07,1.47) | -0.16(-0.62,0.30) | 1.13(0.77,1.49) | -0.20(-0.29,-0.12) |
| Vitamin A deficiency | -13.67(-19.72,-7.62) | 2.85(0.52,5.18) | -0.51(-2.06,1.04) | 4.02(2.82,5.21) | -0.65(-0.94,-0.37) |
| Occupational particulate matter, gases, and fumes | -2.04(-3.76,-0.32) | 2.16(1.58,2.73) | 0.57(0.15,0.99) | 1.70(1.43,1.98) | -0.11(-0.20,-0.03) |
| **High income countries** |  |  |  |  |  |
| High body-mass index | -13.60 (-14.18, -13.04) | -4.71 (-4.88, -4.55) | -3.79 (-3.91, -3.67) | 0.27 (0.17, 0.55) | -1.14 (-1.20, -1.07) |
| Non-exclusive breastfeeding | 42.17 (12.14, 53.87) | 16.65 (5.42, 20.12) | 12.77 (7.97, 16.26) | -0.42 (-6.67, 1.67) | 4.18 (0.84, 13.39) |
| Diet low in calcium | 51.41 (24.86, 73.47) | 17.40 (8.05, 26.28) | 14.79 (-0.88, 21.58) | -0.08 (-3.85, 13.31) | 4.72 (1.38, 21.89) |
| Occupational ergonomic factors | 94.68 (43.19, 99.81) | 59.23 (27.97, 74.10) | 46.72 (19.87, 58.72) | -1.96 (-3.81, 33.66) | 16.19 (1.66, 44.47) |
| Occupational particulate matter, gases, and fumes | 97.18 (83.53, 99.89) | 63.14 (52.83, 68.08) | 52.13 (43.69, 64.79) | -2.67 (-4.28, 5.83) | 15.48 (12.70, 38.49) |
| Household air pollution from solid fuels | 96.60 (81.82, 99.89) | 64.24 (54.98, 73.19) | 51.67 (44.37, 59.34) | -3.63 (-5.99, -2.23) | 14.69 (5.81, 17.45) |
| Diet high in trans-fatty acids | 16.53 (13.51, 18.74) | 5.91 (4.76, 6.72) | 4.73 (4.09, 5.28) | -0.25 (-0.63, 0.77) | 1.38 (1.12, 2.77) |
| Vitamin A deficiency | 69.15 (61.03, 77.75) | 24.04 (20.99, 26.99) | 19.35 (16.93, 21.70) | -1.20 (-1.90, -0.70) | 5.70 (4.88, 6.56) |
| **Upper middle income countries** |  |  |  |  |  |
| High body-mass index | -3.76 (-3.96, -3.57) | -4.83 (-5.19, -4.48) | 0.17 (-0.25, 0.57) | -0.73 (-0.86, -0.47) | -4.23 (-4.66, -3.78) |
| Non-exclusive breastfeeding | 10.55 (9.67, 12.04) | 13.65 (11.51, 14.97) | -0.23 (-1.38, 0.95) | 2.22 (1.85, 4.39) | 11.65 (9.38, 13.51) |
| Diet low in calcium | 2.43 (2.29, 2.57) | 3.13 (2.91, 3.32) | -0.09 (-0.35, 0.20) | 0.49 (0.42, 0.56) | 2.7 (2.40, 3.01) |
| Occupational ergonomic factors | 3.93 (3.69, 4.17) | 5.13 (4.88, 5.38) | -0.13 (-0.55, 0.26) | 0.79 (0.65, 0.91) | 4.44 (3.99, 4.88) |
| Occupational particulate matter, gases, and fumes | 38.53 (21.23, 42.35) | 44.99 (20.14, 56.33) | -4.53 (-8.21, 17.84) | 9.06 (-4.45, 33.17) | 44.75 (21.18, 51.13) |
| Household air pollution from solid fuels | 2.02 (1.81, 2.25) | 2.63 (2.35, 2.90) | 0.05 (-0.17, 0.27) | 0.42 (0.30, 0.48) | 2.15 (1.71, 2.58) |
| Diet high in trans-fatty acids | 4.47 (3.87, 5.08) | 6.22 (5.76, 6.63) | -0.25 (-0.78, 0.45) | 0.97 (0.82, 2.71) | 5.58 (5.25, 5.91) |
| Vitamin A deficiency | 2.91 (2.62, 3.19) | 3.77 (3.40, 4.11) | 0.03 (-0.30, 0.36) | 0.6 (0.46, 0.69) | 3.13 (2.56, 3.68) |
| **Lower middle income countries** |  |  |  |  |  |
| High body-mass index | -3.58 (-5.03, -2.06) | -3.24 (-3.62, -2.89) | -0.82 (-1.04, -0.60) | -0.12 (-0.51, 1.06) | -1.93 (-2.07, -1.77) |
| Non-exclusive breastfeeding | 10.93 (3.49, 16.24) | 7.60 (2.49, 21.2) | 2.23 (-3.35, 13.82) | 1.38 (0.23, 9.34) | 4.55 (1.76, 14.60) |
| Diet low in calcium | 3.31 (2.11, 4.47) | 2.54 (1.91, 3.07) | 0.61 (0.38, 1.46) | 0.38 (0.11, 1.57) | 1.59 (0.91, 3.27) |
| Occupational ergonomic factors | 10.63 (7.68, 12.97) | 6.26 (4.28, 9.09) | 1.25 (0.41, 6.65) | 0.92 (0.36, 3.43) | 4.17 (3.12, 7.36) |
| Occupational particulate matter, gases, and fumes | 53.56 (16.07, 80.27) | 52.10 (-9.13, 86.74) | 23.28 (-14.58, 71.19) | 5.01 (-1.18, 63.29) | 33.32 (0.67, 77.40) |
| Household air pollution from solid fuels | 1.01 (0.55, 1.46) | 1.01 (0.93, 1.09) | 0.26 (0.20, 0.32) | 0.05 (-0.30, 0.16) | 0.58 (0.50, 0.62) |
| Diet high in trans-fatty acids | 2.95 (1.78, 4.05) | 2.61 (2.23, 3.09) | 0.67 (0.42, 0.93) | 0.36 (0.11, 0.48) | 1.63 (1.47, 3.59) |
| Vitamin A deficiency | 0.86 (0.48, 1.22) | 0.84 (0.76, 0.91) | 0.22 (0.16, 0.27) | 0.04 (-0.26, 0.14) | 0.48 (0.41, 0.52) |
| **Low income countries** |  |  |  |  |  |
| High body-mass index | -1.81 (-1.96, -1.64) | -5.51 (-6.46, -4.69) | -0.33 (-0.61, -0.03) | -0.37 (-0.44, 0.24) | -4.77 (-5.34, -4.22) |
| Non-exclusive breastfeeding | 2.41 (2.17, 2.69) | 7.92 (7.30, 8.57) | 0.64 (0.38, 1.07) | 0.54 (-0.26, 0.62) | 6.66 (6.41, 7.37) |
| Diet low in calcium | 1.52 (-0.13, 3.77) | 4.30 (1.30, 6.40) | 0.37 (-4.24, 2.59) | 0.09 (-6.08, 3.93) | 3.67 (2.15, 6.75) |
| Occupational ergonomic factors | 1.57 (1.38, 2.57) | 4.93 (4.10, 5.57) | 0.32 (0.07, 1.34) | 0.30 (-0.19, 0.42) | 4.17 (3.74, 4.70) |
| Occupational particulate matter, gases, and fumes | 22.83 (0.72, 31.23) | 46.19 (17.28, 72.77) | 11.45 (-3.88, 28.62) | 2.10 (-3.39, 34.81) | 36.06 (1.46, 66.03) |
| Household air pollution from solid fuels | 0.49 (0.45, 0.54) | 1.53 (1.34, 1.74) | 0.10 (0.03, 0.17) | 0.10 (-0.07, 0.13) | 1.32 (1.20, 1.45) |
| Diet high in trans-fatty acids | 2.20 (1.18, 4.99) | 7.19 (6.27, 8.86) | 0.55 (-0.08, 2.62) | 0.47 (-0.22, 1.49) | 6.12 (4.61, 13.60) |
| Vitamin A deficiency | 0.43 (0.38, 0.48) | 1.33 (1.12, 1.54) | 0.09 (0.03, 0.15) | 0.08 (-0.06, 0.10) | 1.16 (1.03, 1.30) |

* ASIR: Age-standardized incidence rate; IHD: ischemic heart disease; IS: ischemic stroke; ICH: intracerebral hemorrhage; SAH: subarachnoid hemorrhage.


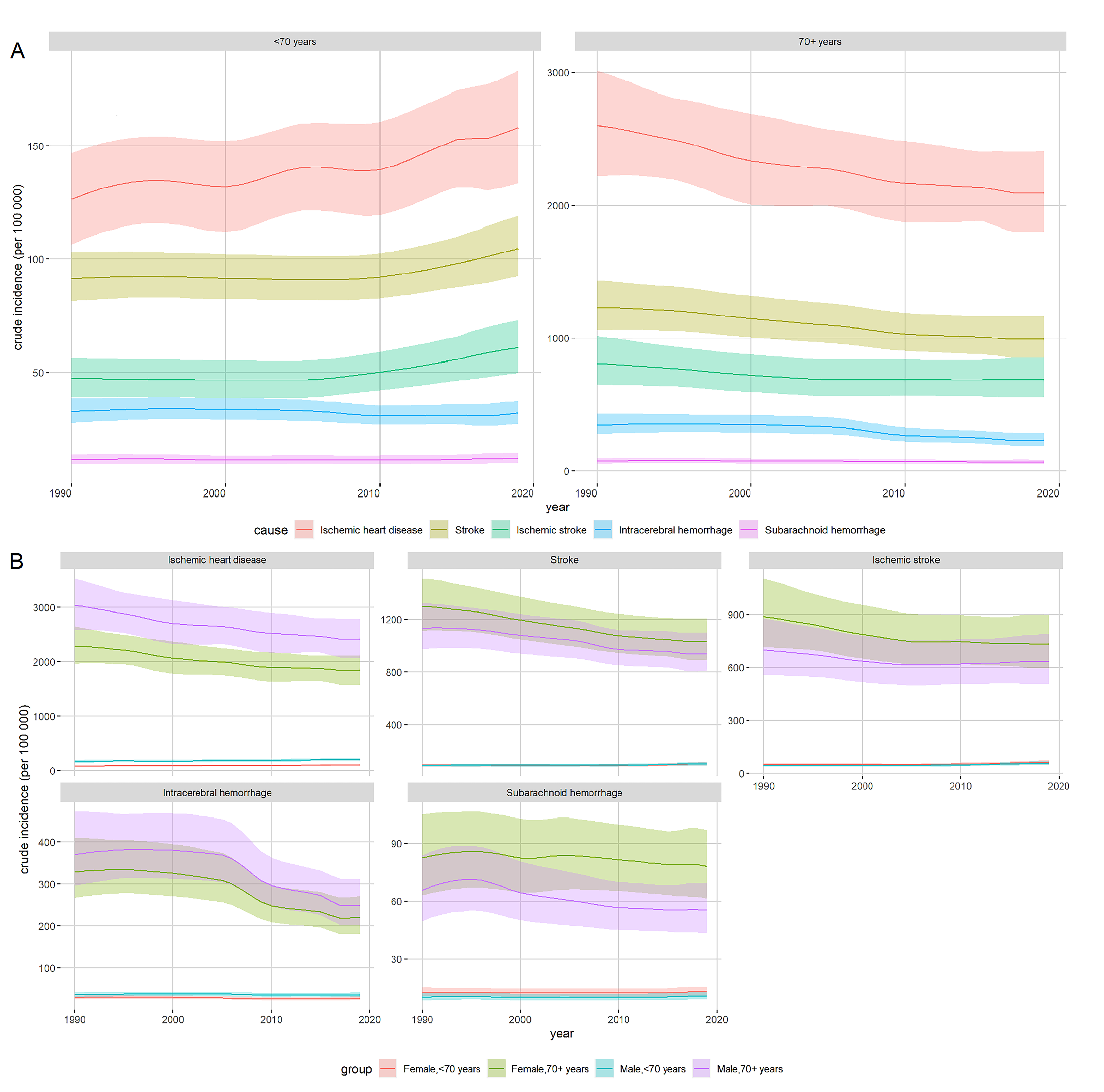


**S1 Fig:** Trends in crude incidence of ischemic heart disease and subtypes of stroke from 1990-2019 in the younger (<70 years) and older populations (≥70 years).


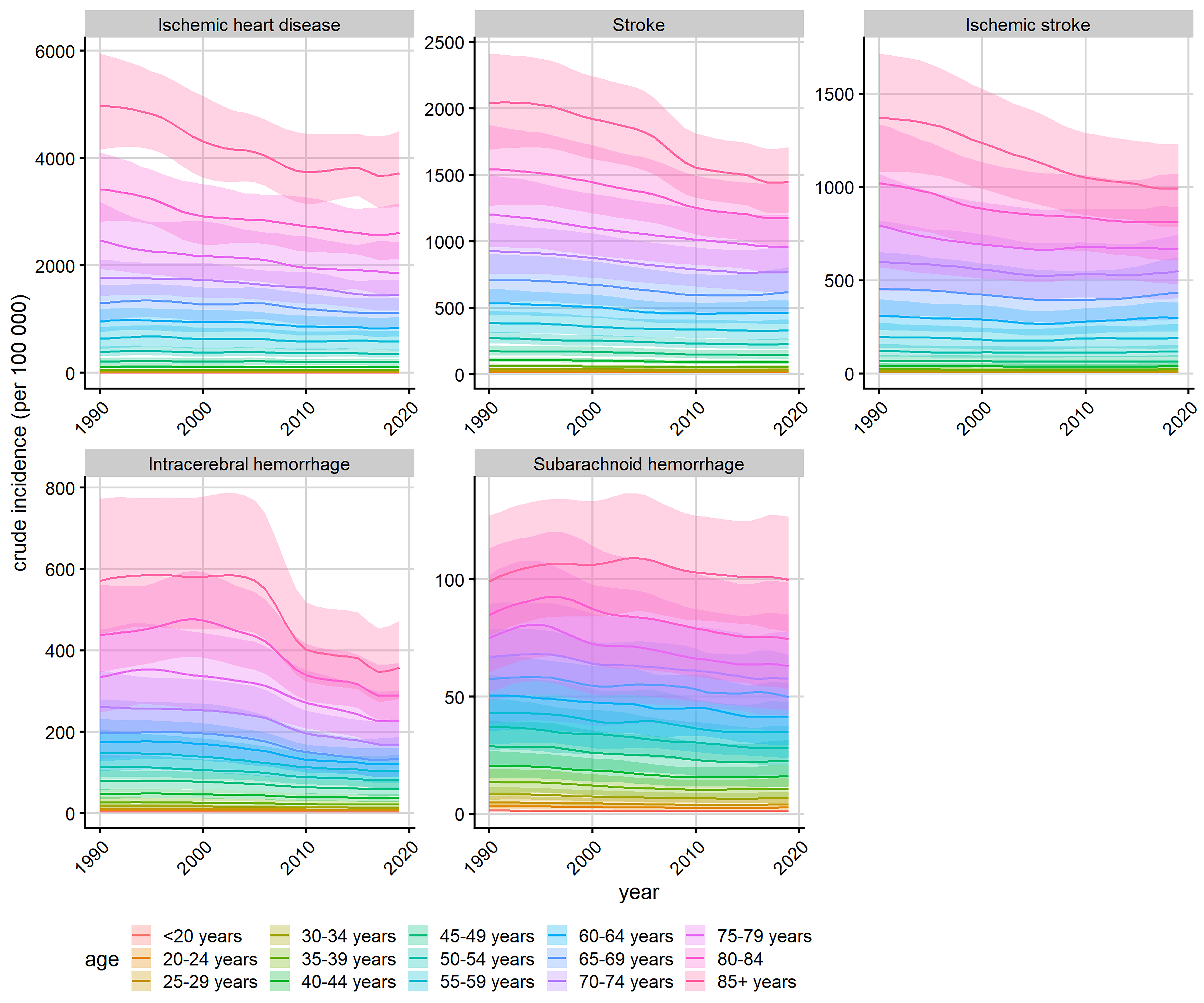


**S2 Fig:** Trends in age-specific incidence of cardiovascular diseases from 1990-2019





**S3 Fig:** Trends in age-standardized incidence of cardiovascular diseases from 1990-2019 by World Bank income levels of countries or territories
